# GraphProt: modeling binding preferences of RNA-binding proteins

**DOI:** 10.1186/gb-2014-15-1-r17

**Published:** 2014-01-22

**Authors:** Daniel Maticzka, Sita J Lange, Fabrizio Costa, Rolf Backofen

**Affiliations:** 1 Department of Computer Science, Albert-Ludwigs-Universität Freiburg, Freiburg im Breisgau, Germany; 2 Centre for Biological Signalling Studies (BIOSS), Albert-Ludwigs-Universität Freiburg, Freiburg im Breisgau, Germany

## Abstract

We present GraphProt, a computational framework for learning sequence- and structure-binding preferences of RNA-binding proteins (RBPs) from high-throughput experimental data. We benchmark GraphProt, demonstrating that the modeled binding preferences conform to the literature, and showcase the biological relevance and two applications of GraphProt models. First, estimated binding affinities correlate with experimental measurements. Second, predicted Ago2 targets display higher levels of expression upon Ago2 knockdown, whereas control targets do not. Computational binding models, such as those provided by GraphProt, are essential for predicting RBP binding sites and affinities in all tissues. GraphProt is freely available at http://www.bioinf.uni-freiburg.de/Software/GraphProt.

## Background

Recent studies have revealed that hundreds of RNA-binding proteins (RBPs) regulate a plethora of post-transcriptional processes in human cells [1-3]. The gold standard for identifying RBP targets are experimental cross-linking immunoprecipitation-high-throughput sequencing (CLIP-seq) protocols [4-6]. Despite the great success of these methods, there are still some problems to overcome: (1) the data may contain many false positives due to inherent noise [7,8]; (2) a large number of binding sites remain unidentified (a high false-negative rate), because CLIP-seq is sensitive to expression levels and is both time and tissue dependent [9] and (3) limited mappability [10] and mapping difficulties at splice sites lead to further false negatives, even on highly expressed mRNAs. To analyze the interaction network of the RBPome and thus to find all binding sites of a specific RBP, a CLIP-seq experiment is only the initial step. The resulting data requires non-trivial peak detection to control for false positives [7,8]. Peak detection leads to high-fidelity binding sites; however, it again increases the number of false negatives. Therefore, to complete the RBP interactome, computational discovery of missing binding sites is essential. The following describes a typical biological application of computational target detection. A published CLIP-seq experiment for a protein of interest is available for kidney cells, but the targets of that protein are required for liver cells. The original CLIP-seq targets may have missed many correct targets due to differential expression in the two tissues and the costs for a second CLIP-seq experiment in liver cells may not be within the budget or the experiment is otherwise not possible. We provide a solution that uses an accurate protein-binding model from the kidney CLIP-seq data, which can be used to identify potential targets in the entire transcriptome. Transcripts targeted in liver cells can be identified with improved specificity when target prediction is combined with tissue-specific transcript expression data. Generating expression data is likely cheaper than a full CLIP-seq experiment.

Computational target detection requires large numbers of highly reliable binding sites for training a binding model. Modern experimental methods such as RNAcompete[3,11] and CLIP-seq[4-6] give a better characterization of RBP-binding specificities due to two important aspects: (1) the number of binding sites available for model training is increased from tens to thousands of sequences and (2) detection of exact binding locations is more precise, ranging from about 30 nucleotides for RNAcompete and high-throughput sequencing of RNA isolated by CLIP (HITS-CLIP) [4] to measurements at the nucleotide level for individual-nucleotide resolution CLIP (iCLIP) [5] and photoactivatable-ribonucleosideenhanced CLIP (PAR-CLIP) [6]. A major qualitative difference between CLIP-seq and RNAcompete data is that the latter determines relative binding affinities *in vitro*, whereas CLIP-seq detects binding events *in vivo*.

There is a clear deficit of computational tools suited to detecting RBP binding sites to date; however, a multitude of sequence-motif discovery tools have been developed to detect DNA-binding motifs of transcription factors [12]. Popular examples are MEME[13], MatrixREDUCE[14] and DRIMust[15]. In the past, some of these methods have also been applied to the analysis of RBP-bound RNAs [16-18].

It has been established that not only sequence, but also structure, is imperative for detecting RBP binding [17,19]. The first tools to introduce structural features into target recognition were BioBayesNet[20] for transcription factor binding sites and MEMERIS[21] for the recognition of RBP targets. MEMERIS is an extension of MEME using RNA accessibility information to guide the search towards single-stranded regions. A recent approach and the current state of the art for learning models of RBP binding preferences is RNAcontext[17,22]. RNAcontext extends accessibility information to include the type of unpaired regions (external regions, bulges, multiloops, hairpins and internal loops). RNAcontext was shown to outperform MEMERIS and a sequence-based approach, MatrixREDUCE, on an RNAcompete set of nine RBPs [17].

Available approaches that introduce a secondary structure into motif detection have two weaknesses. First, a single-nucleotide-based structure profile is used, that is, a nucleotide is considered paired or unpaired (or part of a specific loop). Second, the main assumption behind these models is that nucleotide positions are scored independently. While this assumption seems to work well for RBP motifs located within single-stranded regions, positional dependencies arise when structured regions (that is base-pairing stems) are involved in binding recognition: binding to double-stranded regions involves dependencies between base pairs, which lead to distant stretches of nucleotides in the sequence that can affect the binding affinity [23-27].

The general requirements for accurate binding models are thus manifold. First, training data nowadays comprise several thousands of RBP-bound sequences, therefore, identification of sequence and structure similarities must be computationally efficient. This excludes the use of conventional alignment-based methods (such as LocaRNA [28,29] and RNAalifold [30]). Second, both sequence and structure interdependencies should be modeled, which cannot be achieved by structure-profile-based approaches [17,21,31]. Third, models should be robust with respect to noisy data and be able to take quantitative binding affinities into account.

## Results and discussion

We present GraphProt, a flexible machine-learning framework for learning models of RBP binding preferences from different types of high-throughput experimental data such as CLIP-seq and RNAcompete. Trained GraphProt models are used to predict RBP binding sites and affinities for the entire (human) transcriptome, regardless of tissue-specific expression profiles. We start with a schematic overview of the GraphProt framework and highlight the advantages of this approach. For the first time, in spite of the huge amount of data, we make use of the full secondary structure information by relying on an efficient graph-kernel approach.

We establish that GraphProt has robust and improved performance in comparison to the state of the art by evaluating prediction performance for 24 sets of CLIP-seq and nine sets of RNAcompete data. Prediction performance was clearly improved in comparison to RNAcontext[17,22] and even more clearly in comparison to a sequence-only-based approach, MatrixREDUCE[14], which was added to accentuate the importance of considering secondary structure. To gain further insight into the binding preferences learned by GraphProt models, we devised a procedure to extract simplified sequence and structure binding motifs that could be visualized as well-known sequence logos. We compared our motifs with current data on binding specificities and found substantial agreement.

Finally, we showcase two possible applications that consolidate the biological relevance of GraphProt models. First, we estimated affinities for PTB binding sites when training on CLIP-seq data without access to affinity measurements. As a control, we compared these estimated affinities with additional experimental measurements and observed a significant correlation. Thus, our binding models can learn from simple binding and non-binding information to differentiate between strong and weak binding sites. Second, using a GraphProt model trained on a set of Ago2 HITS-CLIP sites, we verified that predicted Ago2 targets are in agreement with changes in transcript expression levels upon Ago2 knockdown. The same trend was not observed for the original HITS-CLIP-detected sites, clearly indicating that GraphProt identifies binding sites missed by the high-throughput experiment.

### The flexible **GraphProt** framework

**Figure 1:**
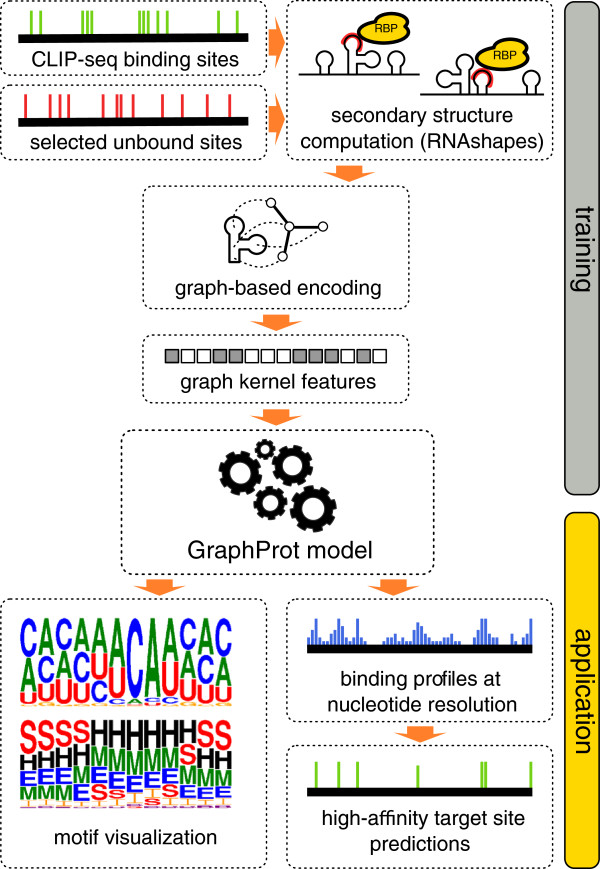
**Schematic overview of the ****GraphProt ****framework.** CLIP-seq, cross-linking and immunoprecipitation sequencing; RBP, RNA-binding protein.

**Figure 2:**
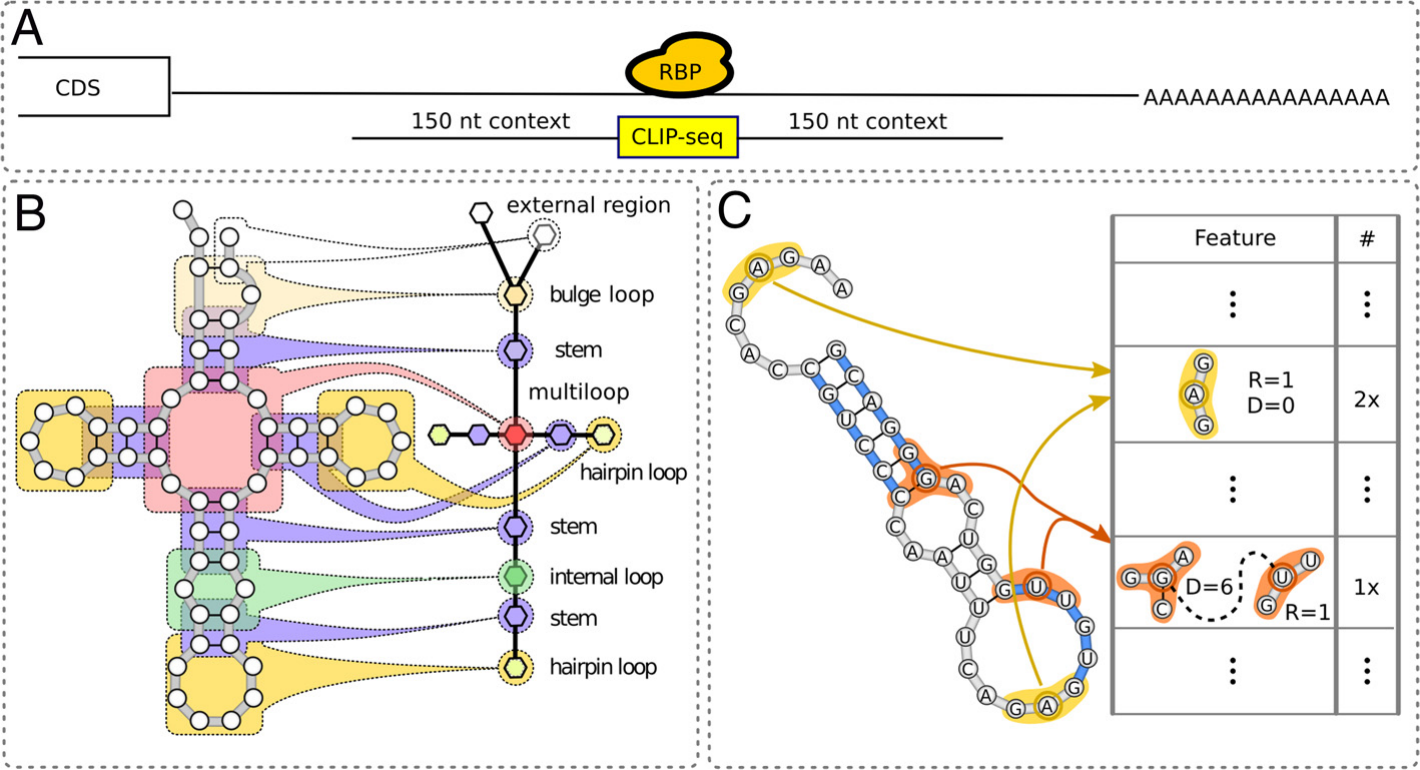
**Natural encoding of RBP-bound sites and graph-kernel features. (A)** The region identified in the CLIP-seq experiment (yellow) is symmetrically extended by 150 nucleotides to compute representative secondary structure information. **(B)** The RNA secondary structure of each RBP-bound context is represented as a graph. Additional information on the type of substructures (that is whether a group of nucleotides is located within a stem or within one of the loop types) is annotated via a hypergraph formalism. **(C)** A very large number of features is extracted from the graphs using a combinatorial approach. A valid feature is a pair of small subgraphs (parametrized by a radius *R*) a small distance apart (parametrized by a distance *D*). The feature highlighted in orange is an example of a feature that can account for the simultaneous interdependencies between sequence and structure information at different locations. CDS, coding sequence; CLIP-seq, cross-linking and immunoprecipitation sequencing; nt, nucleotide; RBP, RNA-binding protein.

The main application of the GraphProt framework is to learn binding preferences using CLIP-seq data and to apply trained models to (1) detect motifs of sequence and structure binding preferences and (2) predict novel RBP target sites within the same organism. Figure 1 presents a schematic outline of the GraphProt framework. There are two main phases, a training and an application phase. In the training phase, RBP binding sites and unbound sites are derived from CLIP-seq data. Highly probable secondary structures (using RNAshapes) are calculated in the context of each potential target site and each structure is encoded as a hypergraph (see Figure 2A) containing both sequence and full secondary structure information. Features are extracted from the hypergraphs using efficient graph kernels. Finally a model is trained using a standard machine-learning approach. In the application phase, the trained models are either (1) processed further to generate sequence and structure logos of learned binding preferences or (2) used in a scanning approach to predict (novel) RBP binding sites. The predictions can be viewed as a profile over the entire transcript from which only high-scoring sites can be selected. Note that when affinity measurements are available for a large set of binding sites, we can train a regression model on these measurements, instead of classifying sites as bound or unbound. In this case affinities are learned and predicted directly. In subsequent results, however, we show that GraphProt can also accurately predict binding affinities when no affinity data are available for training.

In the following, we highlight special features of GraphProt that are not found in RBP-binding prediction tools in the literature.

#### A natural encoding for RNA-binding protein binding sites

Conventional feature encoding in RNA-binding models uses aggregate probabilities per nucleotide to characterize RNA structure, that is, models integrate a structure profile of the bound sequence [17,31,32]. The most common measurement is accessibility, which is the probability that a nucleotide is unpaired [33,34]. Accessibility is used by MEMERIS[21]. In addition, RNAcontext[17] extends accessibility as the probability that an unpaired nucleotide is located within a specific type of loop (for example, a hairpin, bulge or multiloop). These single-nucleotide structure profiles allow encoding of the RBP target sites in sequential data structures, which guarantees higher computational efficiency. The downside of structure profiles is that the original structure information of the RNA molecule is severely compressed: instead of storing exact base-pairing information, only the marginal binding propensity of one nucleotide towards all other nucleotides is considered.

We propose a representation that is more natural and fully preserves base-pairing information (Figure 2). The key idea is to use a small set of stable structures to represent probable folding configurations on the mRNA in the surrounding context of RBP binding sites. These structures are then encoded as graphs with additional annotations for the type of substructure, that is, multiloops, hairpins, bulges, internal loops, external regions and stems (see Figure 2B).

#### Advantages of graph-kernel features

To efficiently process RNA structures encoded as graphs, we propose a method based on graph kernels. The main idea is to extend the *k*-mer similarity for strings (which counts the fraction of common small substrings) to graphs and finally to fit a predictive model using algorithms from the Support Vector Machine (SVM) family [35] for classification problems and Support Vector Regression (SVR) [36] when affinity information is available.

Using a graph-kernel approach, we extract a very large number of features (that is small disjoint subgraphs, see Figure 2C and Materials and methods for details) in a combinatorial manner and assess their importance in discriminating between bound and unbound regions on an mRNA. The use of disjoint subgraphs gives a binding motif that is more expressive than the one offered by traditional position specific scoring matrices [37] because it takes the simultaneous interdependencies between sequence and structure information at different locations into account. Feature importance information can be used, not only to build accurate predictors, but can be subsequently processed to identify sequence and structure binding preferences.

### GraphProt learns binding preferences from **CLIP-seq** data to predict new target sites

Computational approaches for predicting RBP binding sites require large amounts of training data. The current increase in the number of available CLIP-seq data sets make these a valuable data source of target sites bound by specific RBPs. To benchmark the ability of GraphProt to detect binding preferences of RBPs from human CLIP-seq data, we used 24 sets of HITS-CLIP-, PAR-CLIP- and iCLIP-derived binding sites: 23 were curated by doRiNA[38] and an additional set of PTB HITS-CLIP binding sites was taken from [39] (Additional file 1). The Ago1-4 and IGF2BP1-3 sets contain combined binding sites of several proteins; four of the sets consist of ELAVL1 binding sites derived by both HITS-CLIP and PAR-CLIP. Other proteins included are ALKBH5, C17ORF85, C22ORF28, CAPRIN1, EWSR1, FUS, HNRNPC, MOV10, PTB, PUM2, QKI, SFRS1, TAF15, TDP-43, TIA1, TIAL1 and ZC3H7B.

The ability of a computational method to detect RBP target sites is assessed using the well-known tenfold cross-validation technique. The data is subdivided into ten segments. A model of binding preferences is trained on nine segments and target sites are predicted using the remaining segment (see Additional file 2 for details). Results are averaged over ten different train-and-test experiments. This technique assesses the ability of a method to predict RBP target sites that were not seen during training (this is analogous to the prediction of novel sites). The performance is measured as the area under the receiver operating characteristic curve (AUROC).

We compared the performance of GraphProt to RNAcontext[17] and MatrixREDUCE[14]. MatrixREDUCE was added to the benchmark comparison because it is a sequence-based method that previously displayed promising results in a comparison with RNAcontext[17] (the current state of the art). GraphProt uses an extended sequence context for structure prediction, but centers on the CLIP-seq sites using the viewpoint technique (Figure 2A). For a fair comparison, the same context sequences (for structure prediction) and viewpoint information (for target sites) were used by RNAcontext and MatrixREDUCE (see Materials and methods).

**Figure 3:**
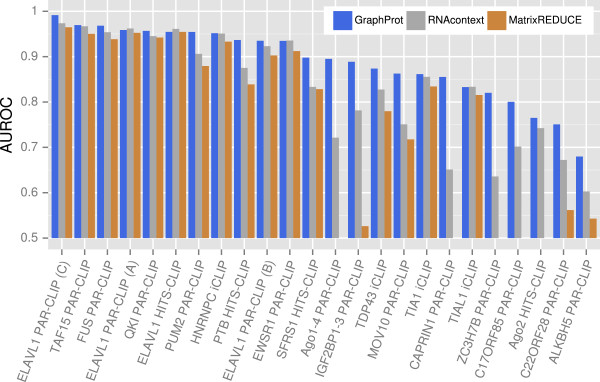
**GraphProt ****performed well in detecting missing binding sites for all RBPs.** Prediction performance was measured using AUROC stemming from a tenfold cross-validation (*y*-axis) on 24 CLIP-seq sets (*x*-axis) for GraphProt, RNAcontext and MatrixREDUCE. GraphProt and RNAcontext consider sequence and structure information, whereas MatrixREDUCE is only sequence based. MatrixREDUCE results below 0.5 are not shown. See Additional file 2 for the full table of results. AUROC, area under the receiver operating characteristic curve; CLIP-seq, cross-linking and immunoprecipitation sequencing; HITS-CLIP, high-throughput sequencing of RNA isolated by cross-linking immunoprecipitation; iCLIP, individual-nucleotide resolution cross-linking and immunoprecipitation; PAR-CLIP, photoactivatable-ribonucleoside-enhanced cross-linking and immunoprecipitation; RBP, RNA-binding protein.

GraphProt outperformed RNAcontext for 20 of the 24 sets, showing an average 29% relative error reduction (Figure 3, Additional file 2). RNAcontext scored only marginally better for the remaining four sets (only a 6% relative error reduction on average). For 11 sets, the improvement in relative error reduction of GraphProt over RNAcontext was over 30%. The largest improvements were a 59% relative error reduction for CAPRIN1 (from AUROC 0.65 to 0.86) and a 62% relative error reduction for AGO1-4 (from AUROC 0.72 to 0.90). Although MatrixREDUCE scored worse than either GraphProt or RNAcontext for all 24 sets, there are some sets where MatrixREDUCE performed nearly as well as the structure-based methods. Nevertheless, it more or less fails for eight data sets. Overall, GraphProt shows robust prediction accuracies and outperforms existing methods.

### GraphProt learns binding preferences from RNAcompete data

The affinity of an RBP to its target site is important for the effectiveness of the subsequent regulation. This implies that a classification into bound and unbound sequences is only a coarse approximation. Instead, a regression approach that can distinguish target sites according to their binding strength is more suitable. To model this binding strength, we require a training set with the affinities for different sequences instead of just a list of bound regions. Such measurements are provided by RNAcompete, an *in vitro* assay used to analyze recognition specificities of RBPs [11]. To measure affinities, a pool of short RNAs, designed to include a wide range of *k*-mers in both structured and unstructured contexts, is exposed to a tagged RBP. The resulting RNA-protein complexes are pulled down and the abundance of bound RNA is measured. Relative binding affinity is then defined as the log ratio between the amount of pull-down RNA and the amount of RNA in the starting pool. Although a modified version of the RNAcompete protocol was published recently [3], the data were not suitable for evaluating GraphProt as the experiment was designed in such a way that it uses only unstructured sequences.

We evaluated the ability of GraphProt to predict binding affinities accurately in a regression setting using the RNAcompete sets for nine RBPs from the initial RNAcompete assay: Vts1p, SLM2, YB1, RBM4, SFRS1, FUSIP1, ELAVL1, U1A and PTB [11]. All sets included both structured and unstructured sequences. The performance of affinity predictions was measured using the mean average precision (APR).

**Figure 4:**
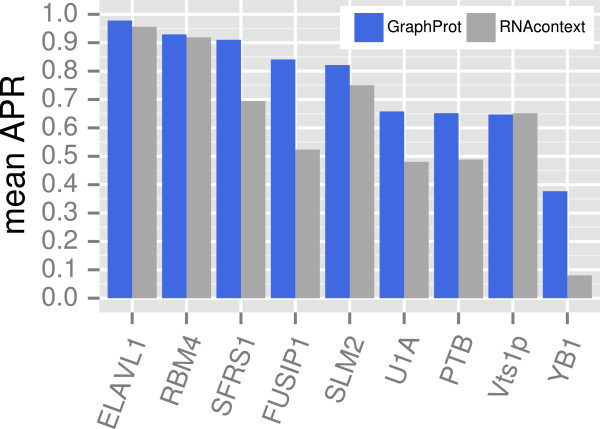
**GraphProt ****uses a regression model to predict binding affinities from measurements derived by ****RNAcompete ****with improved precision.** We present the mean APRs (*y*-axis) for two independent RNAcompete sets (*x*-axis), both comprising nine RBPs, comparing GraphProt and RNAcontext sequence-and-structure-based models. APR, average precision; RBP, RNA-binding protein.

GraphProt outperformed RNAcontext for all proteins except Vts1p, for which RNAcontext scored marginally better (Figure 4, Additional file 2). For five of the proteins, the improvement in relative error reduction was over 30%. The largest improvements in relative error reduction were achieved for FUSIP1 (67%) and SFRS1 (71%). Note that MatrixREDUCE is not shown as previously it did not perform as well as RNAcontext for the exact same data and analysis procedure [17].

### GraphProt models capture known binding preferences

Kernel-based methods allow the use of more complex features and thus an improved prediction performance. On the downside, kernel approaches usually do not provide an insight into what the model has learned. Since this insight is useful for assessing the biological relevance of the CLIP-seq models, we devised a novel post-processing step to identify the sequence and structure preferences learned by the models (see Materials and methods). Note that these logos are a mere visualization aid and do not represent the full extent of the information captured by GraphProt models.

**Figure 5:**
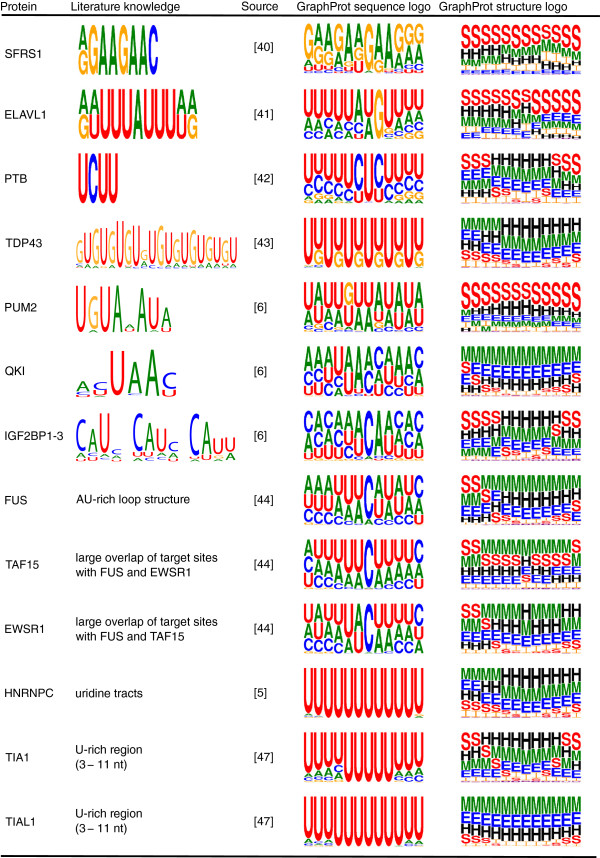
**GraphProt sequence and structure motifs capture known binding preferences.** We compare data from the literature (left) with visualized GraphProt sequence and structure motifs (right) and substantial agreement is evident, especially with known sequence specificities. Structure motifs are annotated with the full set of structure elements: stems (S), external regions (E), hairpins (H), internal loops (I), multiloops (M) and bulges (B). The character size correlates with the importance for RBP binding. For ELAVL1, we show the motif for ELAVL1 PAR-CLIP (C). PAR-CLIP, photoactivatable-ribonucleoside-enhanced cross-linking and immunoprecipitation; RBP, RNA-binding protein.

When compared with data from the literature (Figure 5), we found that GraphProt motifs for SFRS1, ELAVL1 and PTB closely match known SELEX consensus motifs [40-42]. For TDP43, GraphProt identifies a preference for repeated UG dinucleotides. TDP43 targets, determined by RNA immunoprecipitation followed by microarray analysis (RIP-chip), contained such repeats in 80% of the 3^′^ UTRs [43]. GraphProt motifs for PUM2, QKI and IGF2BP1-3 closely resemble the motifs previously identified using the same PAR-CLIP sets [6]. The motifs identified in [6], however, are based on the top sequence read clusters while the GraphProt model was trained using the full sets of PAR-CLIP sites. FUS was found to bind AU-rich loop structures according to electrophoretic mobility shift assays (EMSA) [44]. In accordance with this, the GraphProt structure motif in Figure 5 shows a preference for stems at the borders, but not at the center of the motif. The three members of the FET protein family (FUS, TAF15 and EWSR1) have similar PAR-CLIP binding profiles [44], explaining the stunning similarity of the corresponding GraphProt motifs. Three of the GraphProt motifs (HNRNPC, TIA1 and the closely related TIAL1) show a preference for U-rich sites. HNRNPC was reported to bind to poly-U tracts in 3^′^ and 5^′^ UTRs [5,45,46]. TIA-1 has been described as an ARE-binding protein and binds both U-rich and AU-rich elements. The preference for U-rich regions was shown using SELEX [47], cross-linking and immunoprecipitation [48] and isothermal titration calorimetry (ITC) [49]. Just recently, the high affinity toward binding to U-rich RNA could be traced to six amino acid residues in the TIA1 RNA recognition motif 2 (RRM2)[50].

### RNA structure improves prediction of RNA-binding protein binding

Previous benchmarking analyses (Figures 3 and 4) established that the full GraphProt models (with secondary structure information) are superior to those gained by state-of-the-art methods. Now we assess the importance of secondary structure in RBP binding models. The encoding of RBP target sites is flexible, such that it is easy to remove all structural detail to leave only sequence information. This enables a direct comparison of the full structure to sequence-only models in a controlled setting (that is, the only difference in the comparison is the encoding of the target site). Thus, the added value of structure information for RBP target site prediction can be determined.

**Figure 6:**
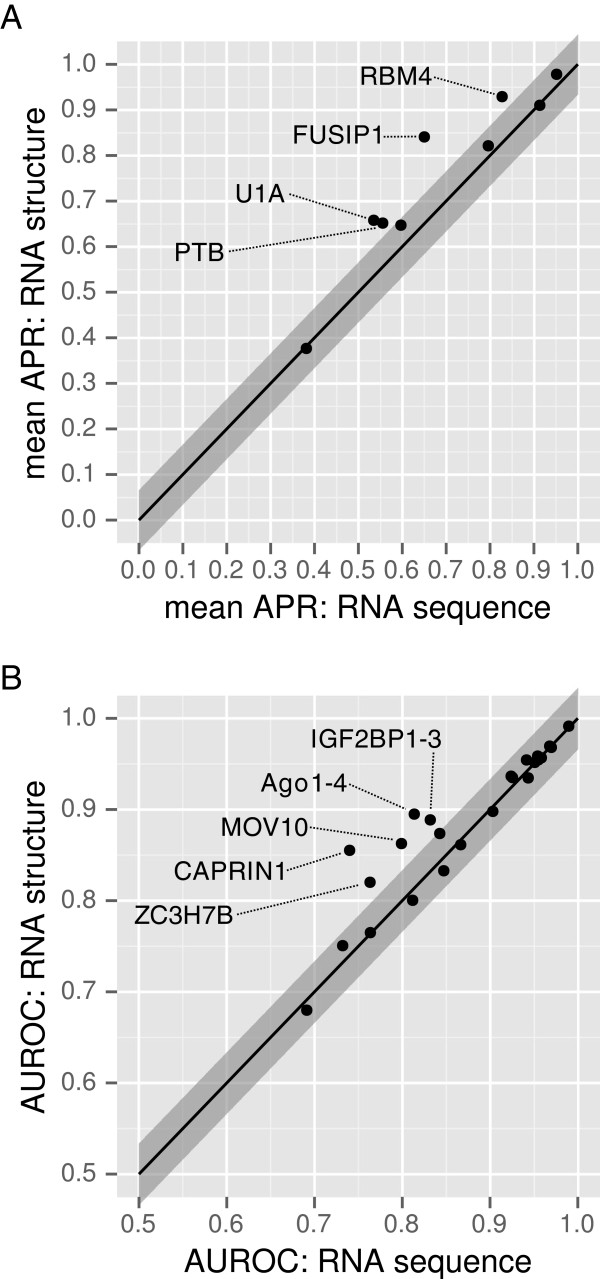
**The difference in predictive power using RNA structure in comparison to sequence-only models.** Full sequence-andstructure models (*y*-axis) and sequence-only (*x*-axis) models were trained on RNAcompete **(A)** and CLIP-seq data **(B)**. The gray ribbons denote the standard deviation of the differences between full structure and sequence-only models. APR, average precision; AUROC, area under the receiver operating characteristic curve; CLIP-seq, cross-linking and immunoprecipitation sequencing.

Both the CLIP-seq and RNAcompete sets (from Figures 3 and 4, respectively) were used to compare models with and without structure information, as shown in Figure 6 (prediction comparisons were performed analogously to previous benchmarking analyses). The average relative error reduction for structure models compared to sequence-only models was 27% for the RNAcompete and 14% for the CLIP-seq sets. The addition of structure improves prediction accuracy in many cases and never leads to a significant loss in performance.

RNAcompete data are optimal for comparing models, since the initial sequences in the library were designed to be either unstructured or to form a stem-loop structure consisting of a single hairpin; therefore, a clear distinction of structure contribution is possible. The results are plotted in Figure 6A. Three of the four proteins from the RNAcompete set showing significant improvements over the sequence models (PTB, RBM4 and U1A) are known to recognize stem-loop structures [51-53]. For PTB, it was determined by ITC, gel shift assays and NMR studies that the two RRM domains bind a stem-loop structure of U1 snRNA [51]. For RBM4, information about possible targets is scarce; however, in one case it was reported that the target of RBM4 is a *cis*-regulatory element that was predicted to be a stem-loop structure [52]. This finding was supported by several mutations that were predicted to disrupt the RNA structure that led to a decreased interaction with RBM4. U1A is also known to bind to a stem-loop structure [53].

**Figure 7:**
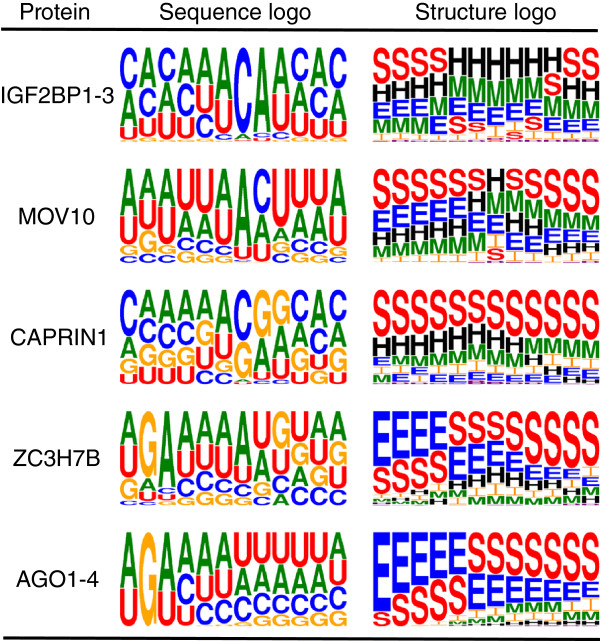
**Sequence and structure motifs for five CLIP-seq sets showing significant improvement of ****GraphProt ****structure over sequence models.** In the visualized logos, the character size determines its importance and structure elements are labeled as follows: stems (S), external regions (E), hairpins (H), internal loops (I), multiloops (M) and bulges (B). All motifs show preferences to both stems and unpaired regions simultaneously. Sequence and structure motifs for Ago1-4 and ZC3H7B are very similar. This can be attributed to the large overlap between ZC3H7B and Ago1-4 PAR-CLIP sites (5,752 of the 28,238 ZC3H7B sites overlap AGO1-4 sites). CLIP, cross-linking and immunoprecipitation; PAR-CLIP, photoactivatable-ribonucleoside-enhanced cross-linking and immunoprecipitation.

In contrast to RNAcompete, CLIP-seq experiments are performed *in vivo* and all of the different types of structure elements could influence binding affinities. Comparisons using the CLIP-seq data are plotted in Figure 6B. For five of the CLIP-seq sets (Ago1-4, CAPRIN1, IGF2BP1-3, MOV10 and ZC3H7B), the performance of the structure models was significantly improved over the sequence models (35% average relative error reduction). The structure motif for IGF2BP1-3 shows a preference for the accessible part of stem-loop structures. Motifs for MOV10, CAPRIN1, ZC3H7B and Ago1-4 indicate preferences for generally structured regions (Figure 7). GraphProt structure models for these proteins also show a higher than average relative error reduction compared to RNAcontext (53% vs 29% average relative error reduction). This indicates that the full RNA structure representations used by GraphProt are better suited than the structure-profile-based approach used by RNAcontext when modeling binding preferences of RBPs binding to structured regions (Additional file 3). Some of the remaining proteins show preferences for structured binding sites in their structure motifs as well as large relative error reductions over RNAcontext, for example, ALKBH5, C17ORF85, C22ORF28, PTB, PUM2, SFRS1 and TDP43. The structure properties of these binding sites may be captured by GraphProt sequence models via dinucleotide frequencies; however, we cannot rule out other reasons for the improved performance of GraphProt sequence models over RNAcontext.

The large-scale analysis of double-stranded RNA-binding proteins (dsRBPs) is slightly lagging behind that of single-stranded RNA-binding proteins (ssRBPs). To the extent of the authors’ knowledge, the first and only genome-wide studies of dsRBPs were performed for MLE, MSL2 (two members of the Male-Specific Lethal complex) [54] and Staufen [55]. The data from these studies, however, is not suitable for training GraphProt models. MLE and MSL2 bind very specifically to only a few sites in the roX1 and roX2 RNAs [54] and for Staufen, only target mRNA was available instead of exact target sites [55]. Therefore, we could not evaluate the performance of GraphProt for dsRBPs binding predominantly to stems; however, the previously mentioned improved performance when studying RBPs binding to mixed structured and accessible regions indicate that GraphProt is well equipped for, and should perform well when, learning binding preferences of dsRBPs.

In summary, for ssRBPs binding to accessible regions, GraphProt sequence models may provide results comparable to the full structure models at increased processing speed. In contrast, the study of proteins binding to structured regions, benefits strongly from the full structure models provided by GraphProt, with larger than average increases in performance over structure-profile-based models. Since full structure models never performed significantly worse than sequence-only models, they should be used as the default.

### Showcase 1: GraphProt learns binding affinities without affinity data

Biologically, it is more important to predict the binding affinity of an interaction than to categorize a potential target site as binding or non-binding. The bottleneck of this computational task is the availability of large data sets of quantitative, experimental measurements of affinities. Although CLIP-seq experiments are becoming increasingly popular, the data from them does not inherently provide a quantification of the binding affinity. In principle, the number of reads mapping to a binding site could be used as a proxy for its affinity, provided there is suitable expression data to normalize read counts. Even if these data exist, which is often not the case, normalization is non-trivial. We therefore ask whether binding affinities can be predicted while learning from only bound vs unbound information, as can be derived from CLIP-seq data.

**Figure 8:**
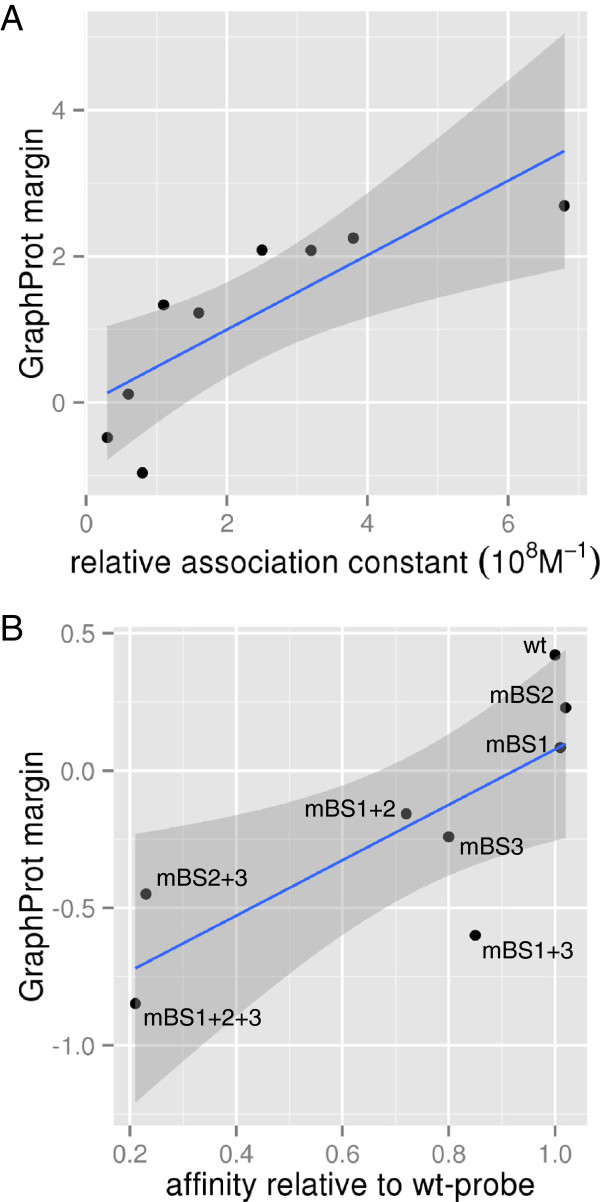
**The certainty of prediction correlates with measured binding affinities.** Prediction certainty is given by GraphProt margins on the *y*-axis and measured affinities for two sets of PTB aptamers on the *x*-axis. Fitted linear models and 95% confidence intervals are depicted in blue and dark gray. Binding affinities are given by **(A)** relative association constants from [42] and **(B)** affinities relative to the wild-type (wt) probe from [56]. wt, wild type.

To test this hypothesis, we compared experimentally derived PTB-binding affinities of two sets of sequences with GraphProt prediction margins using the GraphProt model for PTB HITS-CLIP. Perez and colleagues [42] determined relative affinities from competitive titration experiments for ten sequences of 20 and 31 nucleotides. Karakasiliotis and colleagues [56] identified three PTB consensus sequences starting at positions 112 (BS1), 121 (BS2) and 167 (BS3) of the 5^′^ end of the feline calicivirus genomic RNA and created mutations designed to disrupt PTB binding (mBS1-3) for each site. All combinations of the three modified sites were introduced into probes corresponding to the first 202 nucleotides of the genome, resulting in one wild-type and seven mutant sequences. Affinities were measured using EMSA, so reported affinities are relative to the wild-type probe. We report results for the sequence-only model because the structure model did not show a significant improvement in cross-validation performance over the sequence-only model. For the eight calicivirus probes, we centered on the region containing the three consensus sequences using the viewpoint mechanism. Prediction margins and measured affinities show significant correlation with both sets of sequences (Perez *et al.*: Spearman correlation *r*=0.93, *P*<0.01; Karakasiliotis *et al.*: Spearman correlation *r*=0.76, *P*<0.05). Figure 8 shows prediction margins and reported affinities for both sets. The set of calicivirus probes contains multiple binding sites. Thus, the measured affinities show cooperative effects between binding sites. For example, individual mutations of the first two binding sites (mBS1 and mBS2) slightly increase affinity, but the combined mutation of both sites (mBS1+2) leads to a decreased affinity compared to the wild-type sequence (Figure 8B). Despite the fact that GraphProt does not model cooperative effects, both the wild type as well as the two probes with comparable affinities were assigned positive GraphProt margins while the probes with reduced PTB affinity were predicted to be negative. The only notable outlier is mBS1+3, where GraphProt has overestimated the combined effect of the disrupted PTB consensus sequences.

These results clearly show that, in addition to predicting binding affinities in a regression setting, GraphProt can also be applied to the prediction of binding affinities when only sets of bound sites for a binary classification task are available, as is the case when analyzing CLIP-seq data. This allows the evaluation of putative binding sites with a meaningful score that reflects the biological functionality.

### Showcase 2: Differential expression upon Ago2 knockdown is explained by **GraphProt** predictions but not by published **CLIP-seq** binding sites

**Figure 9:**
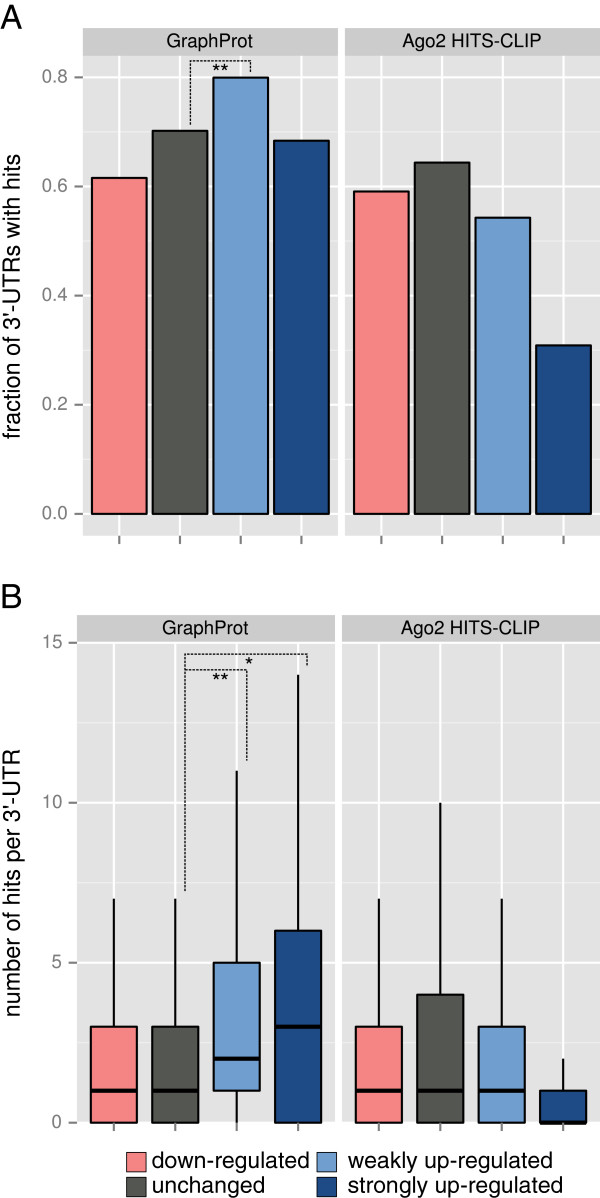
**Targets predicted by the Ago2-HITS-CLIP model are in agreement with measured fold changes after Ago2 knockdown.** Analysis of predicted Ago2 binding events to 3^′^ UTRs that are upregulated after Ago2 knockdown at day 2 for transcripts falling into the following fold-change categories: downregulated (fold change below 0.7, 804 UTRs), unchanged (fold change between 0.7 and 1.4, 6,893 UTRs), weakly upregulated (fold change between 1.4 and 2.0, 713 UTRs) and strongly upregulated (fold change greater than 2.0, 136 UTRs). **(A)** Fraction of 3^′^ UTRs with at least one Ago2 binding site hit. Asterisks indicate a statistically significant increase (*t*-test: * *P*<0.05; ** *P*<0.001). **(B)** Number of binding site hits per 3^′^ UTR. Asterisks indicate a statistically significant increase (Wilcoxon rank sum test: * *P*<0.05; ** *P*<0.001). Box plots do not include outliers, for that reason we show the full distributions in Additional file 4. HITS-CLIP, high-throughput sequencing of RNA isolated by cross-linking immunoprecipitation; UTR, untranslated region.

A typical question in post-transcriptional gene regulation is whether a particular observation can be explained by RBP–RNA interactions. Here, we wanted to explain differential expression upon Ago2 knockdown in comparison to the wild type. Ideally, to obtain RBP target information, a CLIP-seq experiment should be performed for the cell and condition being analyzed, although this is not always feasible. A more economic approach would be to use RBP targets taken from publicly available CLIP-seq data. The problem is that the available data are mostly generated by experiments for other cells or conditions. We showed that publicly available CLIP-seq data do not explain the observed effect, most likely due to differential expression between the two experimental conditions. In contrast, we achieved highly significant agreement when we use GraphProt to detect binding sites missed by a CLIP-seq experiment (Figure 9).

In detail, two independent factors influence the efficiency of downregulating a target mRNA. First, the binding affinity of an RBP to its target site regulates the binding frequency and strength. Second, the number of proteins bound to the same target can increase the signal for subsequent steps in the regulation process [57]. The effect of cooperative regulation when the same element binds multiple times has been especially well studied for Ago2–microRNA interactions [58-61]. Here, Ago2 generally associates with a microRNA and other proteins (together a miRNA-induced silencing complex (miRISC)) to target mRNAs for degradation and/or translational inhibition. A common observation is that several miRISC complexes bind to the same mRNA and the cooperative effect is that the downregulation is stronger [59,61].

In previous work, Schmitter and colleagues established that the mean number of microRNA seed sites per 3^′^ UTR increased significantly between unchanged and weakly upregulated as well as strongly upregulated mRNAs in human HEK293 cells upon Ago2 knockdown [58]. Using their expression data and the same fold-change categories, we investigated the influence of both affinity and cooperative effects based on GraphProt predictions of Ago2 binding sites in comparison to the available CLIP-seq data. The GraphProt sequence-only model was trained on the Ago2-HITS-CLIP set (the use of structure did not improve prediction results for Ago2) and was applied to 3^′^ UTRs with measured fold changes to predict high-scoring target sites.

In showcase 1 (Figure 8), we established that GraphProt prediction margins correlate with measured affinities. Therefore, we estimated high-affinity Ago2 binding sites by only considering the highest-scoring predictions. We compared these predictions to reliable binding sites derived by peak calling on the Ago2-HITS-CLIP read profiles. The overall regulatory effect was investigated by comparing the fraction of 3^′^ UTRs that contain binding sites between the fold-change categories (Figure 9A). An interaction with higher affinity should cause a greater upregulation upon Ago2 knockdown. In a second analysis, cooperative effects were estimated by counting the number of Ago2 binding sites per 3^′^ UTR (Figure 9B) in each fold-change category. For binding sites predicted by GraphProt, both the fraction of 3^′^ UTRs with at least one GraphProt hit (Figure 9A) and the number of GraphProt hits per 3^′^ UTR (Figure 9B) showed a significant increase between unchanged and weakly upregulated transcripts. While there was no major difference in the fraction of UTRs containing UTRs with at least one hit, we saw a clear enrichment for the number of hits in UTRs that are highly regulated, indicating the cooperative effect of multiple miRISC target sites (Figure 9B). In contrast, no correlation was observed for binding sites taken from the Ago2-HITS-CLIP set in both cases (Figure 9).

Since microRNAs guide Ago2 binding, we also looked at computational approaches for detecting microRNA binding sites. To this end, we repeated the analysis from [58] using the same microRNA seeds found to be over-represented in upregulated transcripts and extracted PicTar 2.0 microRNA target predictions from doRiNA[38] to compare against GraphProt (Additional file 4). Both microRNA detection approaches showed some agreement within the differential expression upon Ago2 knockdown; however, the differences between fold-change categories are not as significant in comparison to GraphProt. These results prove the necessity of computational target prediction in addition to performing CLIP-seq experiments. We proved the capacity of GraphProt to predict RBP target sites reliably and even to detect sites missed by experimental high-throughput methods.

## Conclusions

GraphProt is an accurate method for elucidating binding preferences of RBPs and it is highly flexible in its range of application. We used a novel and intuitive representation of RBP binding sites that, in combination with an efficient graph kernel, is able to capture binding preferences of a wide range of RBPs. Depending on the input data, GraphProt models can solve either a regression or a classification task and are thus suitable for learning binding preferences from the two current major sources of experimental data: RNAcompete and CLIP-seq. Trained models are used to predict functional RBP target sites on any transcript from the same organism.

GraphProt had a robust and much improved performance in comparison to the existing state of the art. The full RNA structure representations used by GraphProt were shown to be especially suitable for modeling preferences for binding sites within base-pairing regions. For RBPs known not to be influenced by RNA structure, GraphProt provides very fast sequence-only models that perform as well as the full structure models. RBP sequence and structure preferences learned by GraphProt can be visualized using well-known sequence logos. Beyond the mere elucidation of binding preferences, GraphProt models have been successfully used for diverse tasks such as predicting RBP affinities and scanning for RBP target sites. GraphProt is applicable on a genome-wide scale and can thus overcome the limitations of CLIP-seq experiments, which are time and tissue dependent. We showed that when GraphProt is applied to all transcripts, missing targets are identified in a setting different to the one where the original CLIP-seq experiment was performed.

## Materials and methods

### Graph encoding of RNA sequence and structure

We have proposed an easy-to-adapt method to encode information about RNA sequence and structure in a natural way. The key idea is to use a generic hypergraph formalism to annotate different types of relations: (1) relations between nucleotides, such as sequence backbone or structure base pairs and (2) relations between abstract structure annotations, such as loops or stems, and the corresponding subsequences.

In this paper, we started from the representation used in GraphClust[62], and provide several useful extensions. In GraphClust, an RNA sequence is encoded, together with its folding structure, as a graph, where vertices are nucleotides and edges represent either a sequence backbone connection or a bond between base pairs. We do not require a single best-folding structure (for example, the one achieving minimum free energy) because this is known to be error prone. Instead, we sample the population of all possible structures and retain highly probable, representative candidates. The sampling strategy was implemented via the *shape abstraction* technique introduced by RNAshapes[63]. RNAshapes categorizes all secondary structures according to a simplified representation, called the *shape*, which abstracts certain structural details. Different abstraction levels, which ignore various structure details, are possible, for example, ignoring all bulges, or all bulges and all internal loops. Stem lengths are always ignored. Out of all possible structures that have identical shapes, RNAshapes considers the one with minimum free energy as representative and calls it the *shrep*. We calculated shreps using shifting windows of 150 nucleotides with a step size of 37 nucleotides and predicted up to three shreps that are required to be within 10% of the minimum free energy of the sequence for each window.

In this work, we extended the representation used in GraphClust [62] in three ways: (1) we added a layer of abstract structure information to the secondary structure representation (see Figure 2B); (2) we considered an oriented version of the graphs and (3) we imposed a restriction on the graph, termed the *viewpoint*, so that features are only extracted from the informative part, that is, the part where RBP binding is hypothesized to occur (see Figure 2A).

#### Encoding abstract structure information

**Figure 10:**
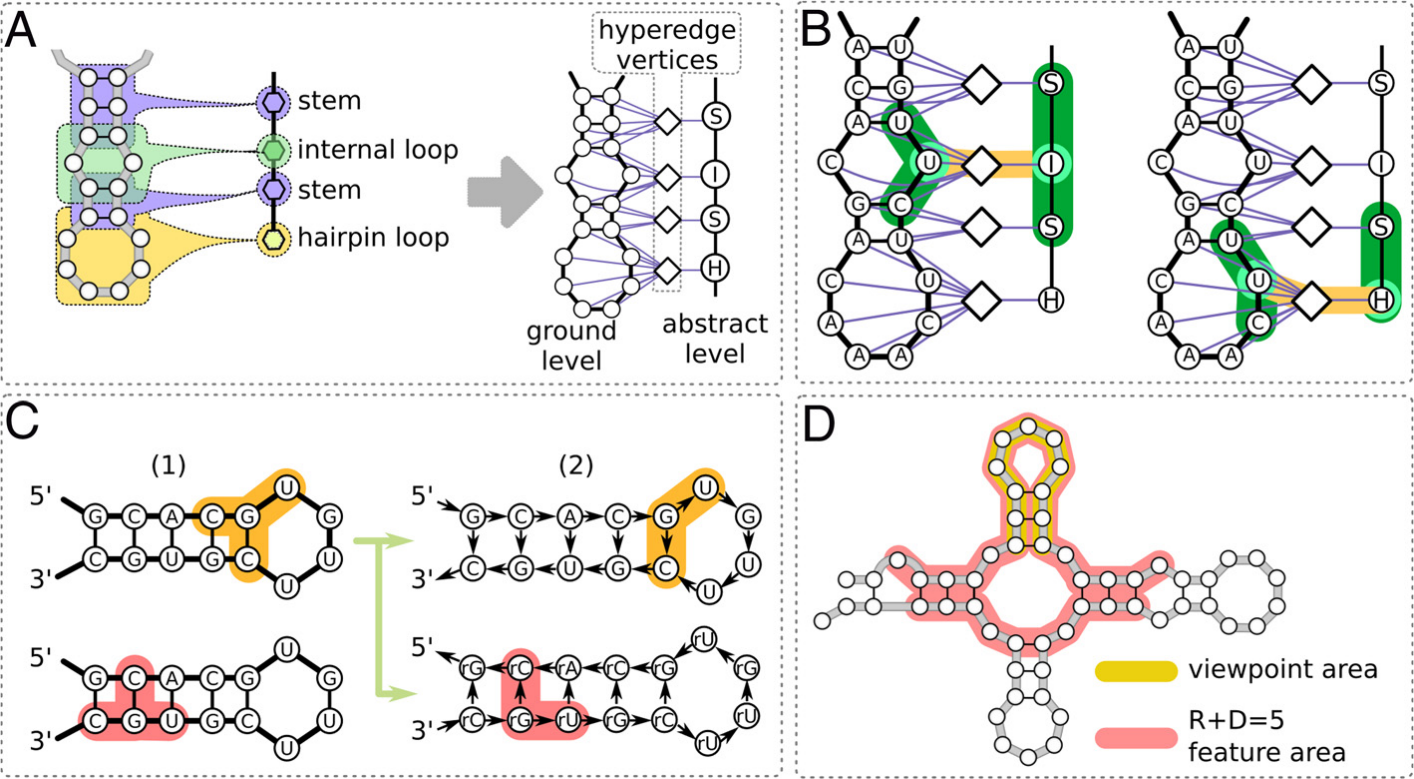
**Extensions to the graph kernel for ****GraphProt****. (A)** Transformation of a hypergraph to an equivalent incident graph. **(B)** Mixed abstract–ground level hypergraph features. Two identical occurrences of the subsequence UUC yield two independent features, one that is aware of the internal loop location and the other that is aware of the hairpin loop location. **(C)** Undirected to directed graph transformation: edges are directed following the 5^′^ to 3^′^ direction. An additional copy of the graph with inverted edges and relabeled vertices (using the prefix *r*) is added. (1) A fragment C(G-C)U is highlighted. In the undirected case, the reversed substructure U(G-C)C generates identical features. (2) The directed treatment creates features that can be used to discriminate between the two fragments. The neighborhood of vertex G generates the feature (G-C)U in the main direction and (*rG*-*r*C)*r*U in the reverse direction. **(D)** Viewpoint extension: a large window allows the RNA molecule to fold correctly; however, as we are interested in a local phenomenon, we restrict the extraction of features to a smaller subportion that reflects the relevant part of the RNA, that is the RBP binding site. We highlighted the viewpoint area in yellow. We highlighted in red the portion of the folded RNA molecule that will be accessed to extract features when the parameters for the NSPD Kernel are radius + distance = 5. RBP, RNA-binding protein.

To model the high-level characteristics of an RNA structure better and to increase the capacity of the model to detect distantly related sequences, we considered an additional layer of secondary structure annotations that we call *abstract*. This layer generalizes the specific nucleotide information and characterizes only the generic shape of a substructure (analogous to the shape abstraction in RNAshapes[63]) such as stems (S), multiloops (M), hairpins (H), internal loops (I), bulges (B) and external regions (E) (see the right-hand side of Figure 2B). This type of annotation is much richer than what could be achieved by merely labeling the corresponding nucleotides (for example, a nucleotide C within a stem could be labeled as C-S and within a bulge loop as C-B) and dependencies can be extracted at a pure abstract level (that is, between abstract secondary structure elements) and at a hybrid level (that is, between abstract secondary structure elements and specific nucleotides). To represent such a rich annotation scheme, we required the expressive power of hypergraphs, which generalize the notion of an edge to that of a relation between many vertices (see Figures 2 and 10).

#### Sequence-only encoding

It is possible to use GraphProt in pure sequence mode, which ignores the RNA secondary structure by discarding base-pairing edges and abstract RNA structures. In this case, GraphProt behaves like an efficient, string kernel machine with gaps in the spirit of [64].

### Graph kernel

The graph kernel used by GraphProt is the Neighborhood Subgraph Pairwise Distance kernel (NSPD Kernel) [65]. In this approach a graph is decomposed into a set of small overlapping subgraphs (see Figure 2C). Every subgraph is then assigned a numerical identifier using an efficient hash-based technique. The identifier is used to solve the isomorphism detection problem in an approximate but extremely fast way and it is used to build the final explicit feature encoding. In this way we build representations that can effectively use millions of features. The type of subgraph chosen in NSPD Kernel is the conjunction of two neighborhood subgraphs at a small distance from each other. Two parameters determine the characteristics of these subgraphs (and are thus related to the complexity and size of the entire feature set): (1) the maximum size of the neighborhood, called the radius *R*, and (2) the maximum distance between any two root nodes, called the distance *D*. Features are extracted for all combinations of values *r*≤*R* and *d*≤*D*.

In this work, the NSPD Kernel was extended in the following way: (1) we upgraded the encoding from graphs to hypergraphs to annotate the RNA abstract structure elements, (2) we considered directed graphs rather than undirected graphs and (3) we introduced a way to select subsets of features using the viewpoint.

#### A kernel for hypergraphs

In the NSPD Kernel of [65], shortest paths can access all vertices and edges in the graph. When the graph contains vertices with a large degree (that is, it is not sparse), however, the shortest path distance becomes degenerate and many vertices are immediate neighbors of each other. Under these conditions, the NSPD Kernel would generate uninformative features corresponding to extremely large subgraphs that are unlikely to occur in more than one instance. Thus, effective learning or generalization would be impossible. This situation would occur if we used the incident graph representation for hypergraphs as shown in Figure 10A (left). Hyperedges (that is, relations) would yield vertices with a large degree. For example, a hairpin loop relation would produce a vertex connected to all nucleotides belonging to the respective hairpin loop. This would effectively remove the nucleotide order of the RNA sequence, since there would exist a shortest path of length two between any two nucleotides in the original hairpin sequence. To deal with this issue, we extended the NSPD Kernel to work on the incident graph as visualized in Figure 10 by (1) considering the relation vertices as non-traversable by paths and (2) creating additional features (that is, pairs of subgraph decompositions), where the root vertices of the two paired neighborhoods are on the two end points of the hyperedge relation (Figure 10B). In intuitive terms, this yields features that are aware of the nucleotide composition of a substructure and, at the same time, of the position of that substructure in the global abstract structure annotation. Consider Figure 10B. Without the abstract structure annotation, the two occurrences of the subsequence UUC would be indistinguishable. With the abstract annotation, we generate two independent features, one that is aware that UUC is located in an internal loop (the vertex labeled I surrounded by two stems), and another feature that is aware that UUC is located in a hairpin loop (the vertex labeled H, preceded by a stem).

By making the relation vertex non-traversable, we have separated the basic from the abstract part of the graph. The NSPD Kernel features in this case can be divided into three separate sets: one set for the basic part, which corresponds to the features used in GraphClust[62], a set of novel features for the abstract part and finally a *hybrid* set of features that relate the nucleotide composition to the abstract part. Note that the features for the abstract part are independent of the exact nucleotide composition of the underlying substructures and therefore allow a better generalization for distantly related RNA sequences.

#### Directed graphs

Using undirected graphs for RNA sequences (as in GraphClust[62]) means that the order imposed by the 5^′^→3^′^ asymmetry is lost. Hence, a sequence and its reversed counterpart (not the complement) would yield the same feature representation. To overcome this limitation, we extended the NSPD Kernel[65] to use directed graphs. For this, we required an unambiguous definition of edge direction: (1) the sequence backbone edges reflect the natural 5^′^→3^′^ direction, (2) the base-pair edges are directed away from the nucleotide closer to the 5^′^ end and towards the nucleotide closer to the 3^′^ end and (3) edges in the abstract part are directed by starting at the sequence ends and traveling from the inner annotations towards the outer limbs, that is, starting from multiloops and ending at hairpin loops. Finally, to capture all relevant information, while still maintaining the consistency with the chosen direction, we duplicated the graph, relabeled all vertices by adding a distinguishing prefix, and reversed the direction of all edges (see Figure 10C).

#### Selection of kernel viewpoints

In the NSPD Kernel[65] of GraphClust[62], all vertices are considered in the generation of features. This is suitable when global RNA sequences are being compared. For RBP binding sites on mRNA, however, only the local target region could be informative and considering all vertices would lead to a substantial amount of noise and decrease the overall predictive performance. Thus, without losing discriminative power, we reduced the number of vertices considered to a fixed subregion of the sequence called the *viewpoint* (see Figures 2 and 10). In a supervised setting, the viewpoint area is selected randomly for negative examples and, for the positive examples, around the region covered by the RBP-bound sequence identified by the respective high-throughput experimental technique. In a genome-wide scanning setting, it would be selected with a *moving window* approach. Note that we cannot simply reduce the graph encoding to fit exactly this reduced area, since in so doing, we would lose the information needed to estimate the folding structure of the mRNA. We require that the root vertex of at least one of the two neighborhoods is localized in the viewpoint area. This way we still allow accurate folding of the mRNA, by considering 150 nucleotides upstream and downstream of the viewpoint [34], but we only select features that are local to the area of interest. The other hyper-parameters of the NSPD Kernel, namely the distance *D* and the radius *R*, determine the area of influence around the putative target region, that is, the portion of the mRNA used to extract relevant information for the discriminative task (see Figure 10D). The viewpoint technique was first introduced in [66].

### Preparation of training and test data

Binding sites for PTB-CLIP [39] were taken from [GEO:GSE19323] (downloaded from the Gene Expression Omnibus [67]). Sites for all other proteins were downloaded from doRiNA[38] (Additional file 1). Binding sites of more than 75 nucleotides were excluded from all training sets. iCLIP sites were extended by 15 nucleotides upstream and downstream. For each set of CLIP-seq sites, we created a set of unbound sites by shuffling the coordinates of bound sites within all genes occupied by at least one binding site, thus enabling the training of models using a binary classification.

To enable accurate prediction of secondary structures [34], we extended the binding sites in both directions by 150 nucleotides or until reaching a transcript end. Core binding-site nucleotides, but not the additional context for folding, were marked as viewpoints. All expansions were done using genomic coordinates.

Secondary structure profiles for RNAcontext were calculated using a modified version of RNAplfold[33] that calculates separate probabilities for stacking base pairs (that is stems), external regions, hairpins, bulges, multiloops and internal loops. Profiles for RNAcontext were calculated using the full sequences. Training and testing were performed on the same core binding sites that were marked as viewpoints for GraphProt. This ensures that RNAcontext still has access to the full sequence context required for structure prediction while providing the same concise binding sites as used by GraphProt. MatrixREDUCE was also evaluated using only the viewpoints.

Next 3^′^ UTRs for Ago2 binding-site predictions were prepared by selecting a non-overlapping set of transcripts with associated fold changes for Ago2 knockdown on day 2, preferring longer over shorter UTRs and with at least 100 but no more than 3,000 nucleotides.

### Benchmarking **GraphProt** models

The predictive performance of GraphProt models trained on CLIP-seq data was evaluated by a tenfold cross-validation. Classification performance is given as the AUROC using the SVM margins as the diagnostic results of classification. GraphProt has three main components: the graph encoding part, the graph kernel feature part and the predictive model part. These are parametrized. The main parameter in the graph encoding part is the abstraction level of the shape category. In the graph kernel feature part, the main parameters are the maximal radius *R* and the maximal distance *D*, which define the neighborhood subgraph features. In the predictive model part during classification, the SVM models were trained using a stochastic gradient descent approach [68] and the main parameters are the number of training epochs and parameter *λ*, which control the trade-off between the fitting accuracy and the regularization strength (Additional files 5 and 6). For the RNAcompete regressions, the main parameters are *c* and *ε*, which control the trade-off between the fitting accuracy and the regularization strength (Additional file 7). The optimal values for all these parameters were determined jointly via a line search strategy. All of the parameters were kept fixed except one, which was chosen for optimization in a round-robin fashion.

Given the amount of computation required for the optimization phase, all GraphProt parameters and RNAcontext motif widths were evaluated on a set of 1,000 sequences or 10% of the available data, whichever was smaller (Additional files 5, 6 and 8). The sequences used to determine the optimal parameter values were then discarded for the cross-validated performance assessment procedure. MatrixREDUCE automatically selects appropriate motif widths during training. For each fold of the MatrixREDUCE cross-validation, we evaluated a single motif, setting max_motif to 1 (Additional file 9). RNAcontext and MatrixREDUCE were trained using values 1/-1 for positive/negative class sequences and using motif widths ranging from 4 to 12 nucleotides.

Model evaluation for the RNAcompete data was essentially as published for RNAcontext[17]. Models were evaluated through converting them to binary-classification tasks using the published thresholds. Classification performance is given as the APR, which is better suited than AUROC for unbalanced classes (which have few bound sequences and many unbound sequences). For each of the nine proteins, models were created for the two independent sets and in each case tested on the corresponding sets. We report the mean score of the two evaluations. GraphProt parameters were determined using subsets of 5,000 training sequences (Additional file 7). Support vector regressions were performed using libSVM[69]. RNAcontext motif widths were determined using all training sequences (Additional file 8).

We report the improvement in predictive performance as the relative error reduction, defined as (*x*^′^-*x*)/(1-*x*) where *x* is the baseline performance and *x*^′^ is the improved performance. The performance is a function with codomain in the interval [ 0,1] and is 1 when the prediction corresponds exactly to the desired target. The (generalized) error is consequently defined as *e*=1-*x*.

### Predicting RNA-binding protein binding sites

A trained GraphProt model is applied to any transcript (or 3^′^ UTRs) to predict (novel) binding sites from the same organism (across-species compatibility may exist, but was not tested). Two options for prediction are available. First, an entire sequence window, representing a potential binding site, is assigned a score that reflects the likelihood of binding. The score is the *prediction margin* as given by the machine-learning software, for example, the SVM. Positive values indicate a true binding site and negative values indicate that no binding occurs. Second, to generate *prediction profiles* on a nucleotide level, we process the prediction margins reported by the software per feature (that is, the importance of that feature for predicting RBP binding), not per window. Profiles are calculated per nucleotide by summing over all features for which the corresponding nucleotide is a root (central) node (in the feature, that is subgraph, Figure 2C). High-affinity binding sites can be extracted from prediction profiles as we exemplified for Ago2.

#### Prediction of Ago2 target sites

To predict Ago2 target sites, we calculated binding profiles for the 3^′^ UTRs of genes with corresponding fold changes from the Ago2 knockdown experiment in [58] using the GraphProt sequence-only model, trained on the Ago2 HITS-CLIP set. Since proteins do not only bind to single nucleotides, binding scores were averaged for all 12-mer windows. To gain high-affinity Ago2 binding sites we considered the 1% highest-scoring 12-mers and merged overlapping and abutting sites.

### Logos of sequence and structure binding preferences

To provide visual representations for both sequence and structural preferences encoded by the GraphProt models, we predicted and scored the approximately 25,000 folding hypotheses of up to 2,000 CLIP-seq-derived binding sites. For each folding hypothesis per binding site, we extracted only the highest-scoring 12-mer, where the score is the average prediction margin per nucleotide from the binding profile, analogous to the method of predicting the Ago2 binding sites. To visualize structure preferences, we compressed full secondary structure information into structure profiles. A nucleotide is assigned to the structure element it occurs in: stem (S), external region (E), hairpin (H), internal loop (I), multiloop (M) or bulge (B). The 1,000 highest-scoring 12-mer nucleotide sequences and structure profiles were converted into sequence and structure logos, respectively (using WebLogo[70]; all logos are in Additional file 10).

### Availability

The GraphProt software, models, parameters and sequences (CLIP-seq sequences used for training, and PTB and 3^′^ UTR sequences used for predictions) are available for download [71]. GraphProt is included as Additional file 11 for archival purposes.

## Abbreviations

APR: average precision; AUROC: area under the receiver operating characteristic curve; CDS: coding sequence; CLIP: cross-linking and immunoprecipitation; dsRBP: double-stranded RNA-binding protein; EMSA: electrophoretic mobility shift assay; HITS-CLIP: high-throughput sequencing of RNA isolated by cross-linking immunoprecipitation; iCLIP: individual-nucleotide resolution cross-linking and immunoprecipitation; ITC: isothermal titration calorimetry; miRISC: miRNA-induced silencing complex; NMR: nuclear magnetic resonance; NSPDK: Neighborhood Subgraph Pairwise Distance Kernel; PAR-CLIP: photoactivatable-ribonucleoside-enhanced cross-linking and immunoprecipitation; RBP: RNA-binding protein; RIP-chip: RNA immunoprecipitation followed by microarray analysis; RRM: RNA recognition motif; SELEX: Systematic Evolution of Ligands by Exponential Enrichment; seq: sequencing; snRNA: small nuclear RNA; SVM: Support Vector Machine; SVR: Support Vector Regression; ssRBP: single-stranded RNA-binding protein; UTR: untranslated region.

## Competing interests

The authors declare that they have no competing interests.

## Authors’ contributions

DM and RB conceived the project and designed its overall goals. DM prepared the data sets, conducted the experiments and developed the GraphProt motif representation. SJL implemented the classification of abstract RNA structure elements and a significant part of the graph encoding. FC developed the NSPD Kernel and subsequent enhancements. RB researched the literature on RBP binding preferences and conceived of the Ago2 knockdown evaluation. DM, SJL, FC and RB wrote the manuscript. All authors read and approved the final manuscript.

## Supplementary Material

Additional file 1 Source publications of CLIP-seq sets (PDF). Click here for file

Additional file 2 **CLIP cross-validation and RNAcompete validation results (PDF).** The file contains results of the CLIP cross-validations and RNAcompete evaluations (AUROC and APR), estimated predictive performance using tenfold cross-validation, receiver operating characteristic curves for the CLIP cross-validations and precision-recall curves for the RNAcompete evaluations. Click here for file

Additional file 3 **Binding to double-stranded regions (PDF).** Binding to double-stranded regions depends on distant stretches of nucleotides involved in the base pairing. Click here for file

Additional file 4 **Additional analyses for Ago2 binding sites (PDF).** Full distributions of Ago2 binding site hits corresponding to Figure 9B and additional analyses on microRNA target prediction corresponding to Figure 9A,B. Click here for file

Additional file 5 Parameters fitted for GraphProt CLIP-seq sequence models (CSV). Click here for file

Additional file 6 Parameters fitted for GraphProt CLIP-seq structure models (CSV). Click here for file

Additional file 7 Parameters fitted for GraphProt RNAcompete models (CSV). Click here for file

Additional file 8 Motif lengths chosen for RNAcontext models (CSV). Click here for file

Additional file 9 Motif lengths chosen for MatrixREDUCE models (CSV). Click here for file

Additional file 10 **GraphProt motifs for CLIP-seq models (PDF).**GraphProt structure motifs including simplified profiles distinguishing only paired and unpaired positions. Click here for file

Additional file 11 GraphProt version 1.0.1 (ZIP). Click here for file
