# Supplementary material for: GraphProt: modeling binding preferences of RNA-binding proteins
Source: Genome Biol. 2014 Jan 22;15(1):R17. doi: 10.1186/gb-2014-15-1-r17 (PMC4053806; doi:10.1186/gb-2014-15-1-r17)
Supplement: Additional file 1 — Source publications of CLIP-seq sets (PDF). [file gb-2014-15-1-r17-S1.pdf]

Source Publications for CLIP-seq sets

sets downloaded from doRiNA[Anders et al., 2012]

The doRiNA database is available at http://dorina.mdc-berlin.de. We used binding sites for hg19 located at http://dorina.mdc-berlin.de/rbp_browser/download_hg19.html. Additional information on the preparation of the individual tracks is available via the doRiNA web frontend at http://dorina.mdc-berlin.de/rbp_browser/hg19.html.

- Ago2 HITS-CLIP [Kishore et al., 2011]
- ELAVL1 PAR-CLIP (A) & HITS-CLIP [Kishore et al., 2011]
- ELAVL1 PAR-CLIP (B) [Lebedeva et al., 2011]
- ELAVL1 PAR-CLIP (C) [Mukherjee et al., 2011]
- HNRNPC iCLIP [Konig et al., 2010]
- MOV10 PAR-CLIP [Sievers et al., 2012]
- SFRS1 CLIP-seq [Sanford et al., 2009]
- TDP-43 iCLIP [Tollervey et al., 2011]
- TIA1 & TIAL1 iCLIP [Wang et al., 2010]
- EWSR1, FUS & TAF15 PAR-CLIP [Hoell et al., 2011]
- Ago1-4, IGF2BP1-3, PUM2 & QKI PAR-CLIP [Hafner et al., 2010]
- ALKBH5, C17ORF85, C22ORF28, CAPRIN1, ZC3H7B PAR-CLIP [Baltz et al., 2012]

other sets

- PTB HITS-CLIP [Xue et al., 2009],[GSE19323]

References

[Anders et al., 2012] Anders, G., Mackowiak, S. D., Jens, M., Maaskola, J., Kuntzagk, A., Rajewsky, N., Landthaler, M., and Dieterich, C. (2012). doRiNA: a database of RNA interactions in post-transcriptional regulation. *Nucleic Acids Res*, 40(Database issue):D180–6.

[Baltz et al., 2012] Baltz, A. G., Munschauer, M., Schwanhausser, B., Vasile, A., Murakawa, Y., Schueler, M., Youngs, N., Penfold-Brown, D., Drew, K., Milek, M., Wyler, E., Bonneau, R., Selbach, M., Dieterich, C., and Landthaler, M. (2012). The mRNA-bound proteome and its global occupancy profile on protein-coding transcripts. *Mol Cell*, 46(5):674–90.

[Hafner et al., 2010] Hafner, M., Landthaler, M., Burger, L., Khorshid, M., Hausser, J., Berninger, P., Rothballer, A., Ascano, M. J., Jungkamp, A.-C., Munschauer, M., Ulrich, A., Wardle, G. S., Dewell, S., Zavolan, M., and Tuschl, T. (2010). Transcriptome-wide identification of RNA-binding protein and microRNA target sites by PAR-CLIP. *Cell*, 141(1):129–41.

[Hoell et al., 2011] Hoell, J. I., Larsson, E., Runge, S., Nusbaum, J. D., Duggimpudi, S., Farazi, T. A., Hafner, M., Borkhardt, A., Sander, C., and Tuschl, T. (2011). RNA targets of wild-type and mutant FET family proteins. *Nat Struct Mol Biol*, 18(12):1428–31.

[Kishore et al., 2011] Kishore, S., Jaskiewicz, L., Burger, L., Hausser, J., Khorshid, M., and Zavolan, M. (2011). A quantitative analysis of CLIP methods for identifying binding sites of RNA-binding proteins. *Nat Methods*, 8(7):559–64.

[Konig et al., 2010] Konig, J., Zarnack, K., Rot, G., Curk, T., Kayikci, M., Zupan, B., Turner, D. J., Luscombe, N. M., and Ule, J. (2010). iCLIP reveals the function of hnRNP particles in splicing at individual nucleotide resolution. *Nat Struct Mol Biol*, 17(7):909–15.

[Lebedeva et al., 2011] Lebedeva, S., Jens, M., Theil, K., Schwanhausser, B., Selbach, M., Landthaler, M., and Rajewsky, N. (2011). Transcriptome-wide analysis of regulatory interactions of the RNA-binding protein HuR. *Mol Cell*, 43(3):340–52.

[Mukherjee et al., 2011] Mukherjee, N., Corcoran, D. L., Nusbaum, J. D., Reid, D. W., Georgiev, S., Hafner, M., Ascano, M. J., Tuschl, T., Ohler, U., and Keene, J. D. (2011). Integrative regulatory mapping indicates that the RNA-binding protein HuR couples pre-mRNA processing and mRNA stability. *Mol Cell*, 43(3):327–39.

[Sanford et al., 2009] Sanford, J. R., Wang, X., Mort, M., Vanduyn, N., Cooper, D. N., Mooney, S. D., Edenberg, H. J., and Liu, Y. (2009). Splicing factor SFRS1 recognizes a functionally diverse landscape of RNA transcripts. *Genome Res*, 19(3):381–94.

[Sievers et al., 2012] Sievers, C., Schlumpf, T., Sawarkar, R., Comoglio, F., and Paro, R. (2012). Mixture models and wavelet transforms reveal high confidence RNA-protein interaction sites in MOV10 PAR-CLIP data. *Nucleic Acids Res*, 40(20):e160.

[Tollervey et al., 2011] Tollervey, J. R., Curk, T., Rogelj, B., Briese, M., Cereda, M., Kayikci, M., Konig, J., Hortobagyi, T., Nishimura, A. L., Zupunski, V., Patani, R., Chandran, S., Rot, G., Zupan, B., Shaw, C. E., and Ule, J. (2011). Characterizing the RNA targets and position-dependent splicing regulation by TDP-43. *Nat Neurosci*, 14(4):452–8.

[Wang et al., 2010] Wang, Z., Kayikci, M., Briese, M., Zarnack, K., Luscombe, N. M., Rot, G., Zupan, B., Curk, T., and Ule, J. (2010). iCLIP predicts the dual splicing effects of TIA-RNA interactions. *PLoS Biol*, 8(10):e1000530.

[Xue et al., 2009] Xue, Y., Zhou, Y., Wu, T., Zhu, T., Ji, X., Kwon, Y.-S., Zhang, C., Yeo, G., Black, D. L., Sun, H., Fu, X.-D., and Zhang, Y. (2009). Genome-wide analysis of PTB-RNA interactions reveals a strategy used by the general splicing repressor to modulate exon inclusion or skipping. *Mol Cell*, 36(6):996–1006.
