# Supplementary material for: GraphProt: modeling binding preferences of RNA-binding proteins
Source: Genome Biol. 2014 Jan 22;15(1):R17. doi: 10.1186/gb-2014-15-1-r17 (PMC4053806; doi:10.1186/gb-2014-15-1-r17)

CLIP-seq crossvalidation results

Dataset	GraphProt		RNAcontext		MatrixReduce	
	PR	ROC	PR	ROC	PR	ROC
ALKBH5 PAR-CLIP	0.669	0.680	0.585	0.600	0.527	0.537
C17ORF85 PAR-CLIP	0.775	0.800	0.670	0.695	0.377	0.303
C22ORF28 PAR-CLIP	0.746	0.751	0.676	0.671	0.518	0.545
CAPRIN1 PAR-CLIP	0.851	0.855	0.635	0.650	0.415	0.352
Ago2 HITS-CLIP	0.756	0.765	0.715	0.732	0.381	0.264
ELAVL1 HITS-CLIP	0.940	0.955	0.943	0.958	0.938	0.954
SFRS1 HITS-CLIP	0.898	0.898	0.842	0.833	0.837	0.828
HNRNPC iCLIP	0.947	0.952	0.947	0.951	0.926	0.933
TDP43 iCLIP	0.895	0.874	0.864	0.828	0.813	0.780
TIA1 iCLIP	0.842	0.861	0.837	0.855	0.807	0.817
TIAL1 iCLIP	0.819	0.833	0.819	0.833	0.804	0.815
Ago1-4 PAR-CLIP	0.906	0.895	0.730	0.721	0.406	0.275
ELAVL1 PAR-CLIP (B)	0.935	0.935	0.918	0.923	0.895	0.903
ELAVL1 PAR-CLIP (A)	0.951	0.959	0.953	0.962	0.942	0.952
EWSR1 PAR-CLIP	0.942	0.935	0.936	0.935	0.901	0.912
FUS PAR-CLIP	0.970	0.968	0.953	0.954	0.931	0.938
ELAVL1 PAR-CLIP (C)	0.992	0.991	0.972	0.974	0.950	0.957
IGF2BP1-3 PAR-CLIP	0.901	0.889	0.792	0.778	0.539	0.519
MOV10 PAR-CLIP	0.853	0.863	0.715	0.750	0.682	0.717
PUM2 PAR-CLIP	0.958	0.954	0.917	0.906	0.887	0.879
QKI PAR-CLIP	0.971	0.957	0.964	0.945	0.959	0.942
TAF15 PAR-CLIP	0.973	0.970	0.969	0.967	0.942	0.950
PTB HITS-CLIP	0.925	0.937	0.863	0.875	0.828	0.839
ZC3H7B PAR-CLIP	0.813	0.820	0.613	0.636	0.435	0.405

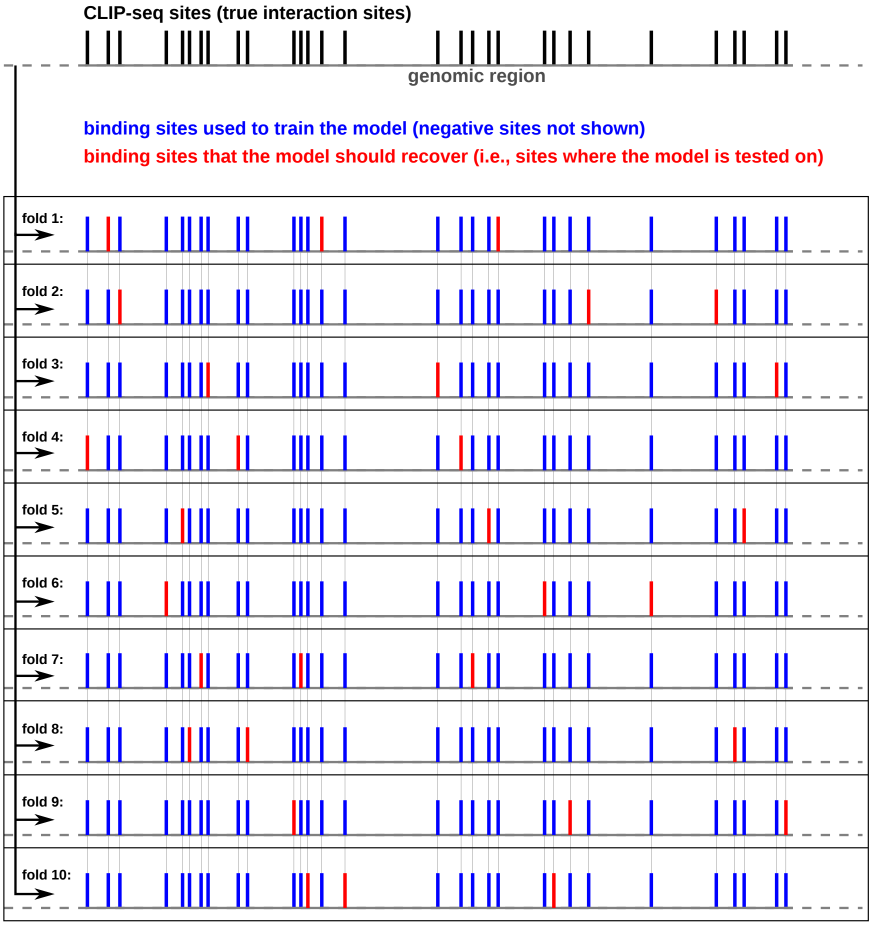

Figure 1: **Estimating predictive performance using 10-fold crossvalidation.** To estimate the performance of a predictive model, two sets of data are needed. The predictive model is trained on the first data set (called training set), the predictive performance of this model is then evaluated using the second data set (called test or validation set). In order to estimate how the predictive model will generalize from the training data (e.g. how well it will predict binding sites for new sequences not part of the training data), no sequence should be part of both datasets. When high-quality data for training and evaluating predictive models is scarce, as is usually the case with RBP binding sites, the predictive performance of a model can be estimated – using only a single dataset – via cross-validation. The data, in this case the set of bound and unbound sites derived from CLIP-seq, is subdivided into ten segments (hence 10-fold crossvalidation). A model of binding preferences is trained on nine segments and target sites are predicted using the remaining segment. This train-and-test procedure is repeated 10 times, using each of the 10 segments as a validation set once. To get a single performance score, the results are averaged over all ten train-and-test experiments.

RNAcompete validation results

Dataset	GraphProt		RNAcontext	
	PR	ROC	PR	ROC
Fusip	0.841	0.983	0.515	0.895
ELAVL1	0.978	0.999	0.832	0.994
PTB	0.379	0.892	0.390	0.906
RBM4	0.928	0.996	0.773	0.984
SFRS1	0.909	0.993	0.671	0.927
SLM2	0.820	0.990	0.550	0.976
U1A	0.657	0.935	0.478	0.871
VTS1	0.647	0.949	0.577	0.956
YB1	0.374	0.897	0.057	0.661

ROC and Precision-Recall Curves

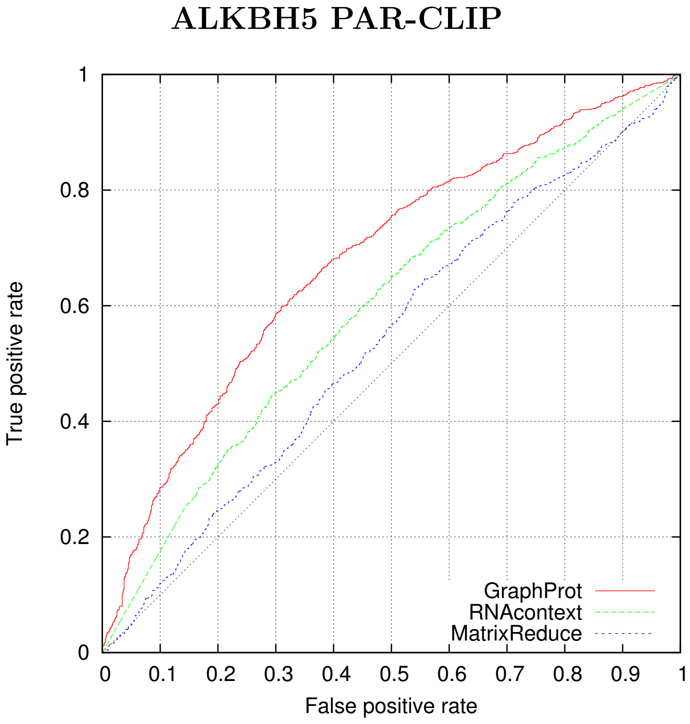

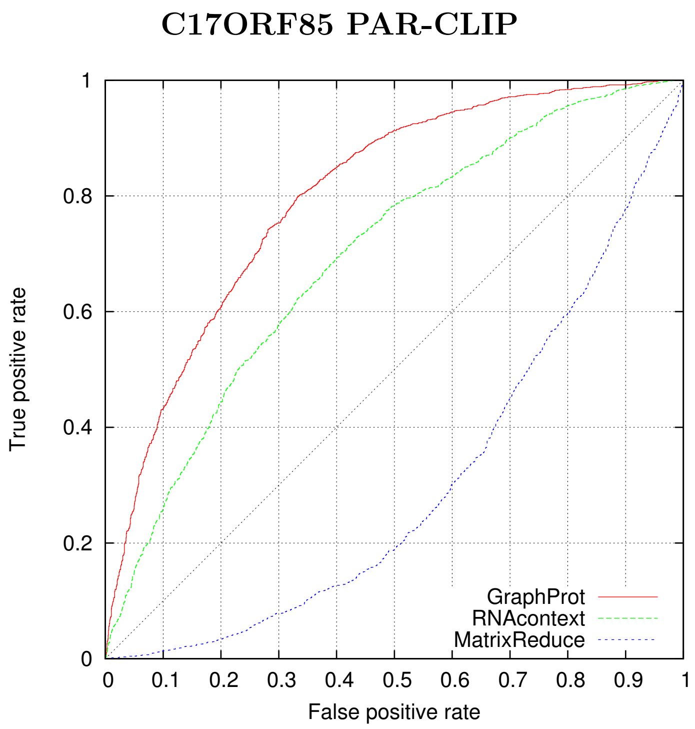
C17ORF85 PAR-CLIP
True positive rate
False positive rate
0
0.2
0.4
0.6
0.8
1
0
0.1
0.2
0.3
0.4
0.5
0.6
0.7
0.8
0.9
1
GraphProt
RNAcontext
MatrixReduce

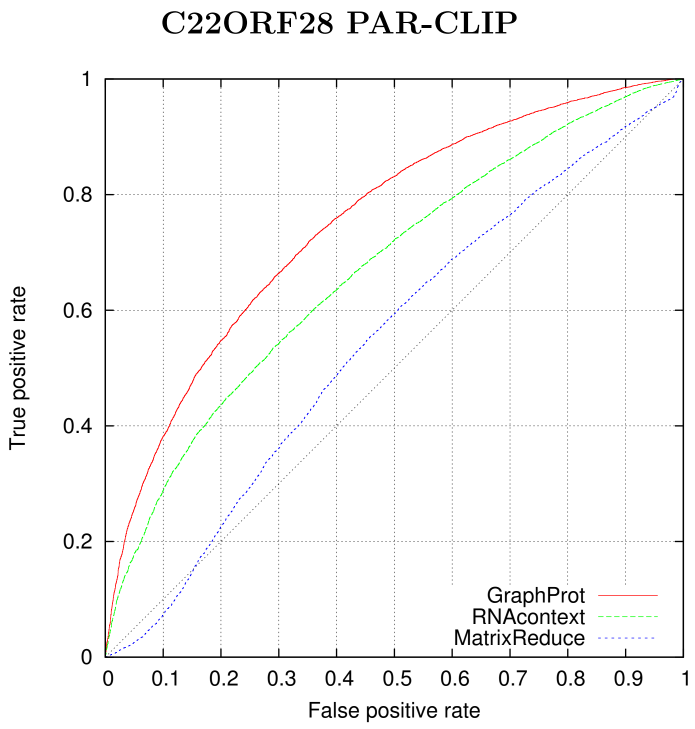
C22ORF28 PAR-CLIP
True positive rate
False positive rate
0
0.2
0.4
0.6
0.8
1
0
0.1
0.2
0.3
0.4
0.5
0.6
0.7
0.8
0.9
1
GraphProt
RNAcontext
MatrixReduce

CAPRIN1 PAR-CLIP

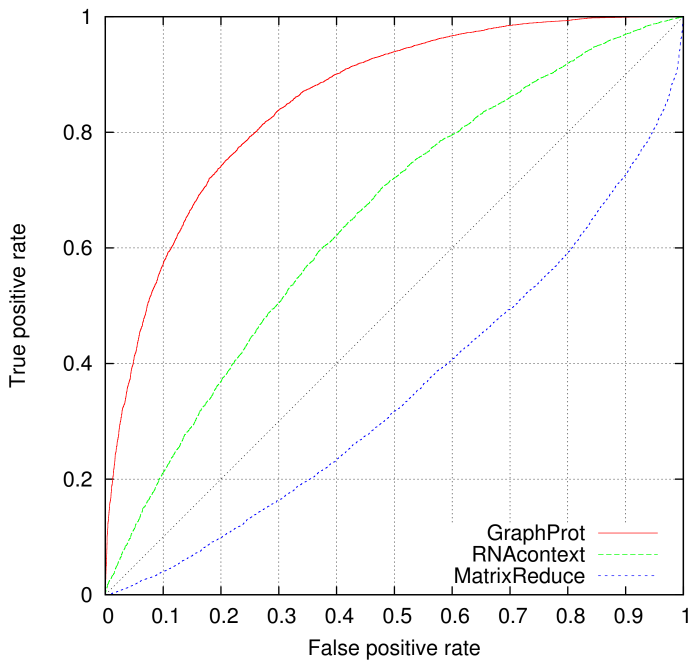

Ago2 HITS-CLIP

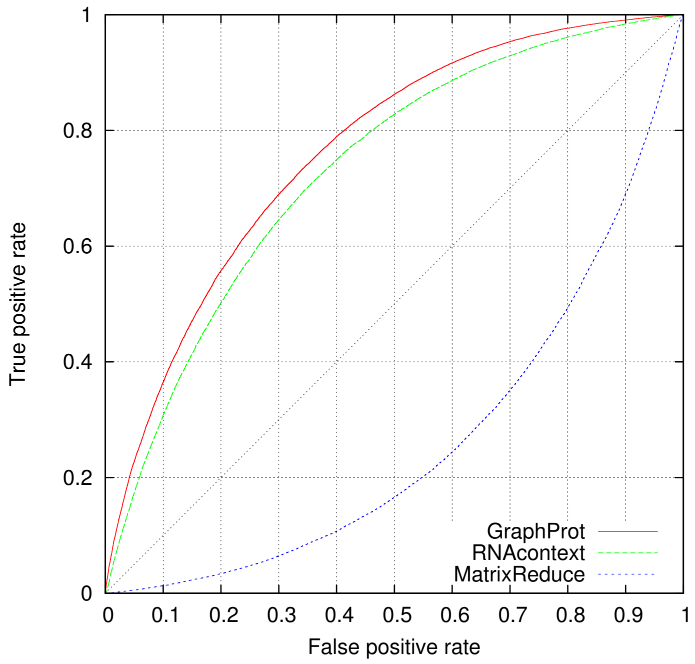

ELAVL1 HITS-CLIP

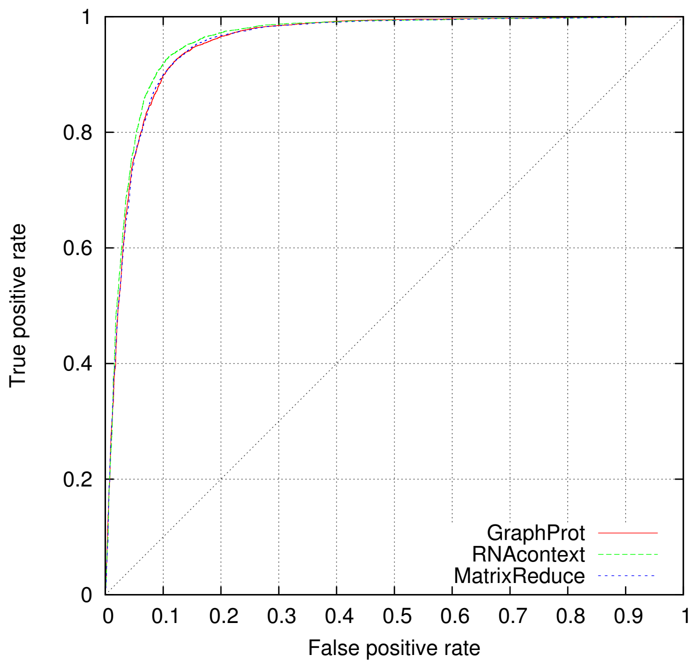

SFRS1 HITS-CLIP

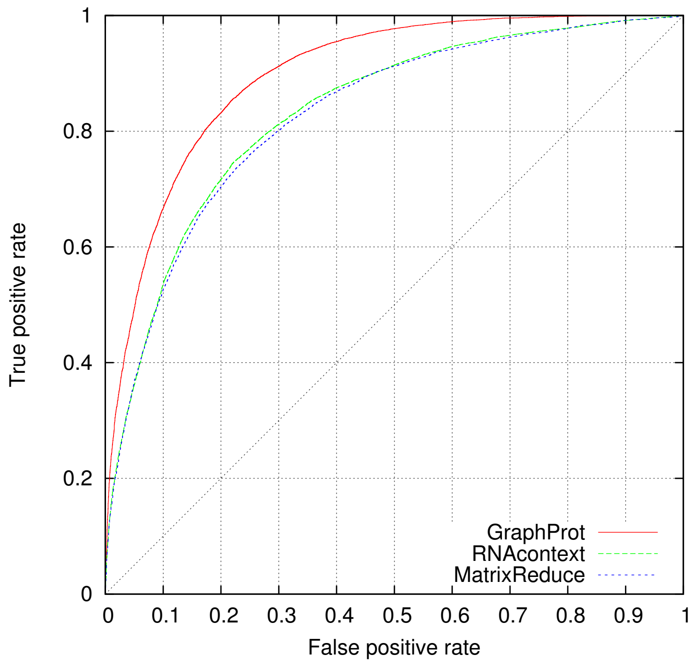

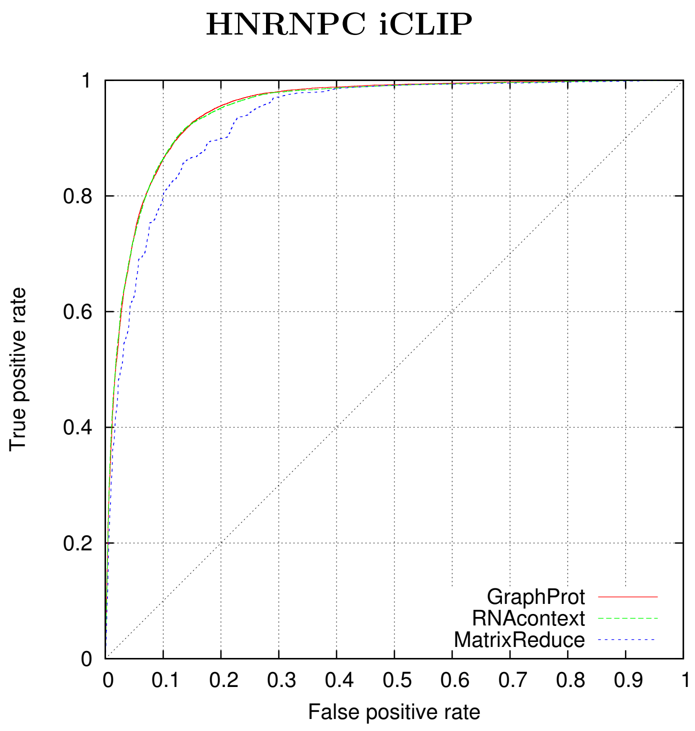
HNRNPC iCLIP
True positive rate
False positive rate
0
0.1
0.2
0.3
0.4
0.5
0.6
0.7
0.8
0.9
1
0
0.2
0.4
0.6
0.8
1
GraphProt
RNAcontext
MatrixReduce

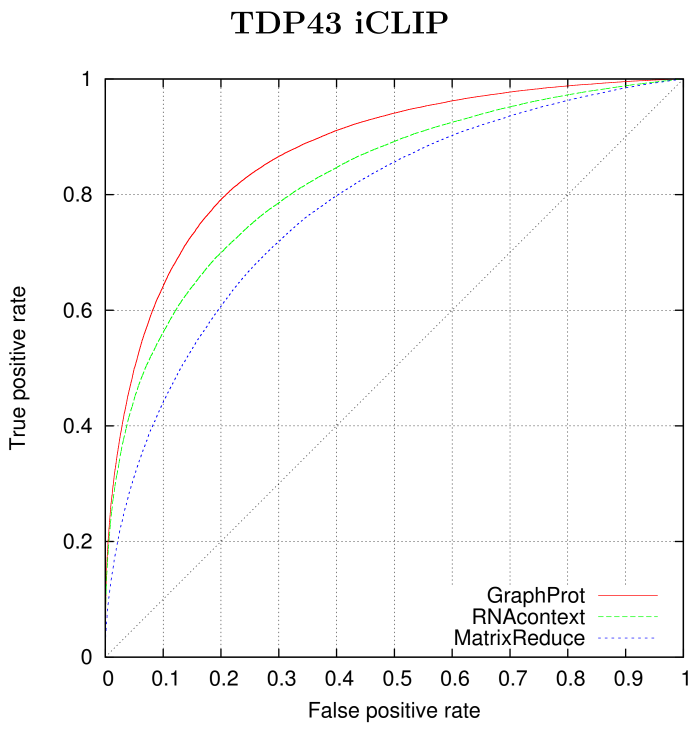
TDP43 iCLIP
True positive rate
False positive rate
0
0.1
0.2
0.3
0.4
0.5
0.6
0.7
0.8
0.9
1
0
0.2
0.4
0.6
0.8
1
GraphProt
RNAcontext
MatrixReduce

TIA1 iCLIP

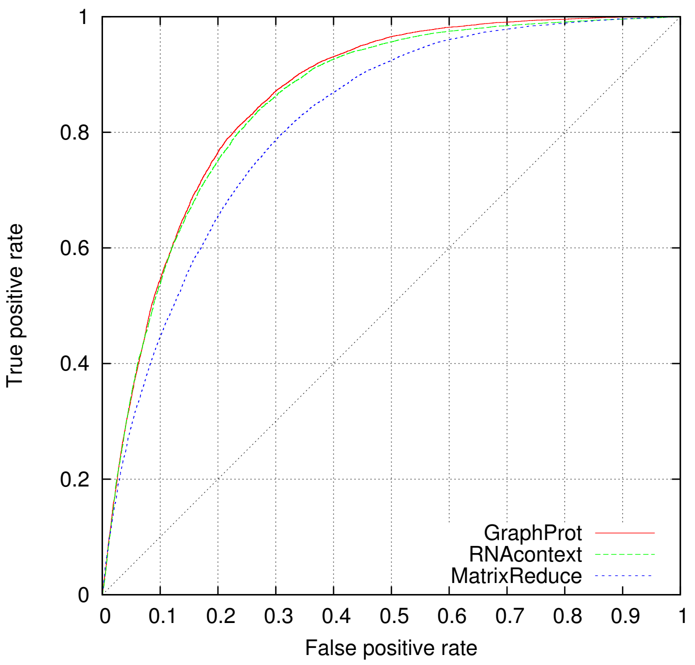

TIAL1 iCLIP

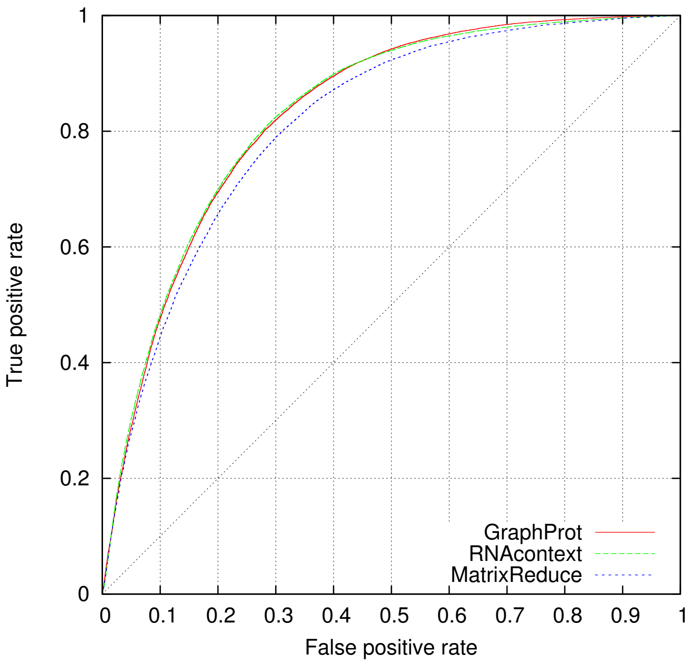

Ago1-4 PAR-CLIP

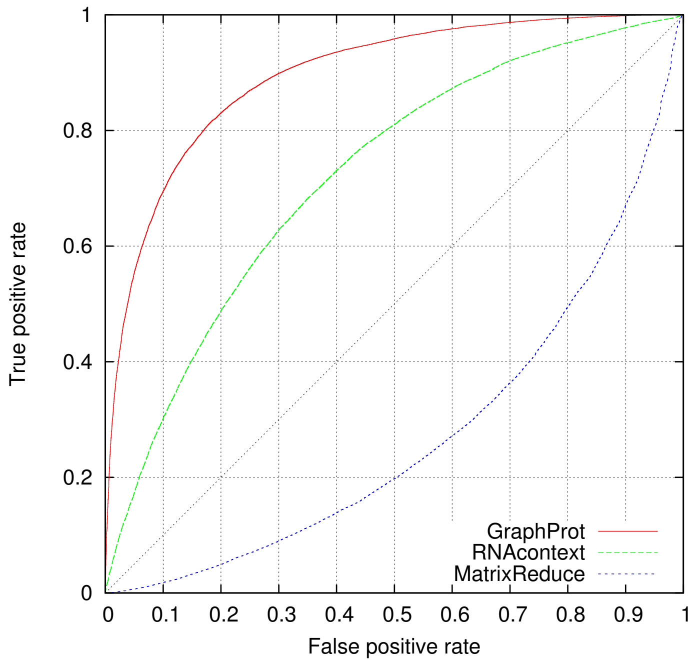

ELAVL1 PAR-CLIP (A)

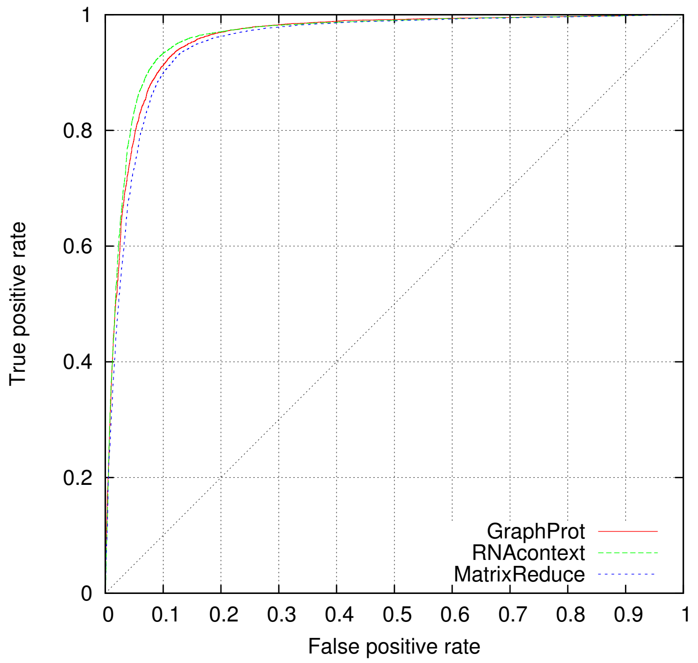

ELAVL1 PAR-CLIP (B)

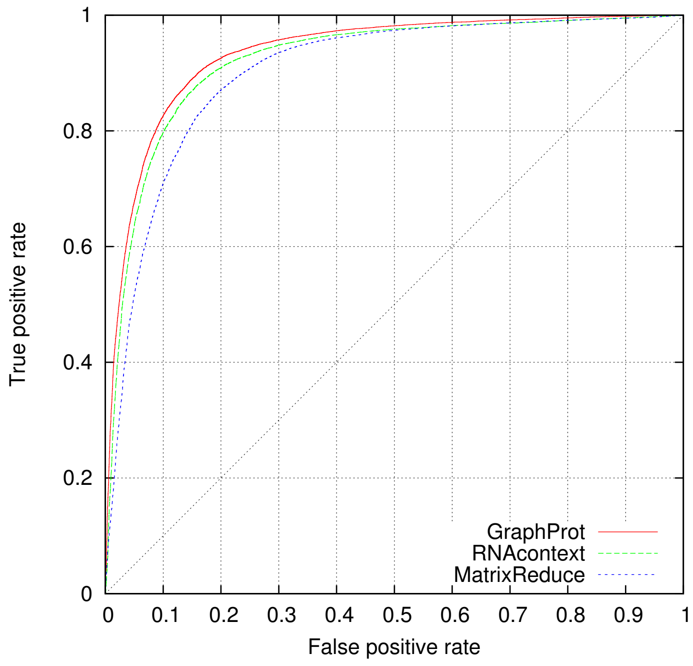

EWSR1 PAR-CLIP

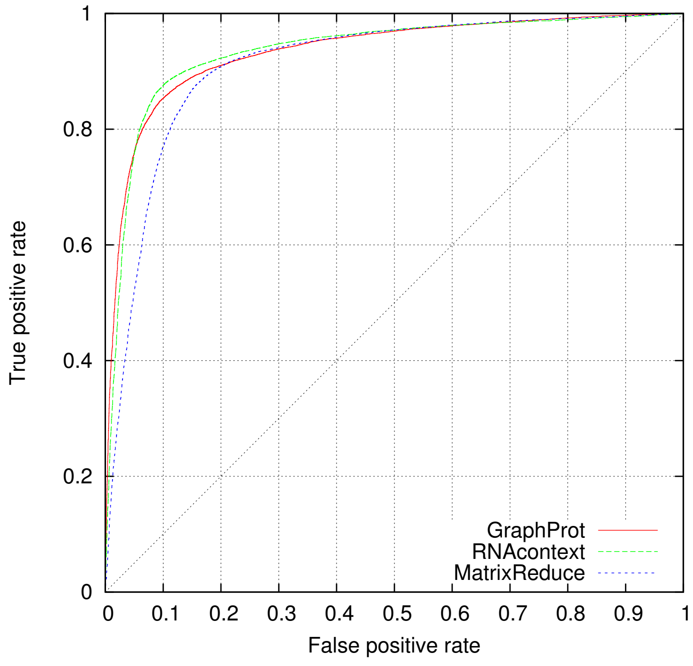

FUS PAR-CLIP

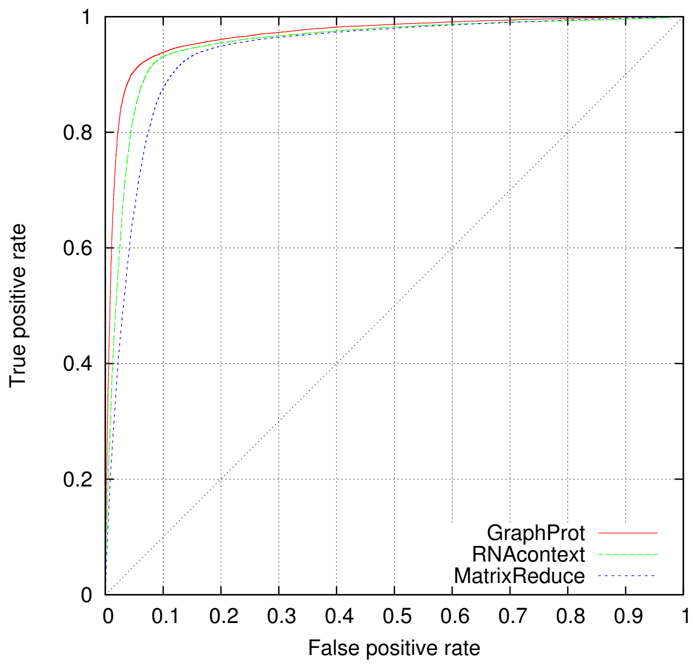

ELAVL1 PAR-CLIP (C)

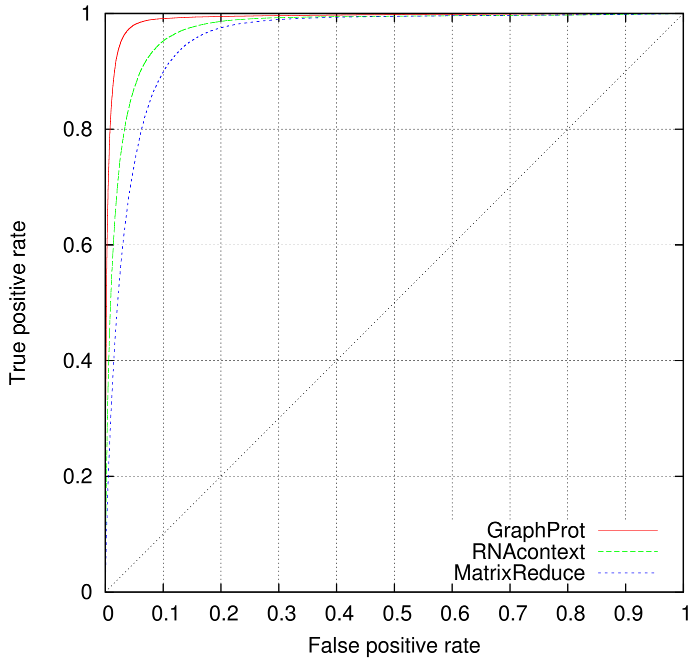

IGF2BP1-3 PAR-CLIP

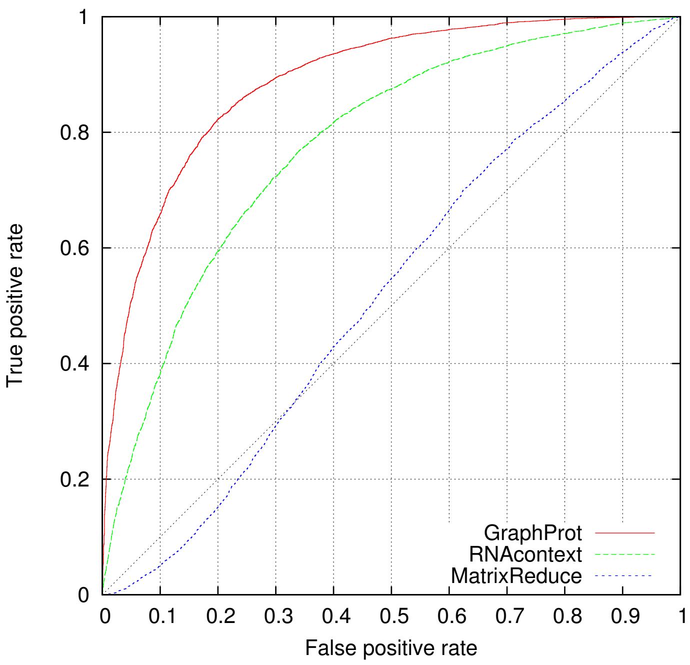

MOV10 PAR-CLIP

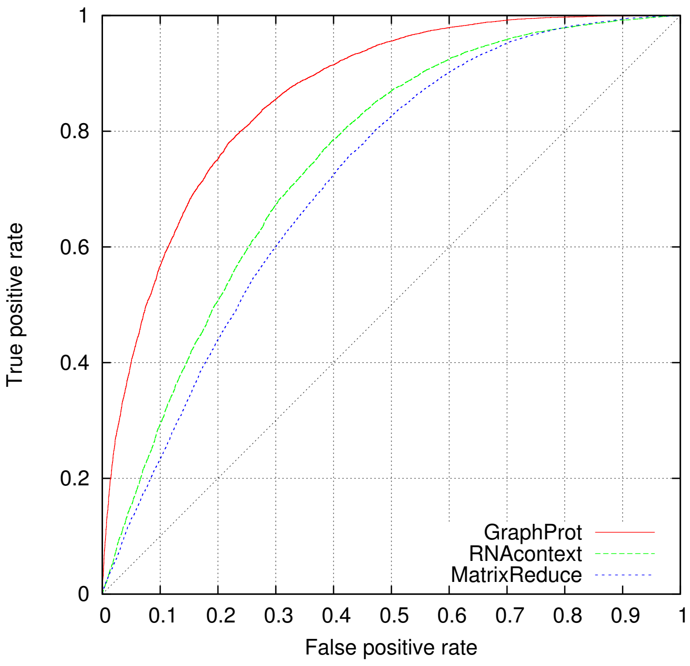

PUM2 PAR-CLIP

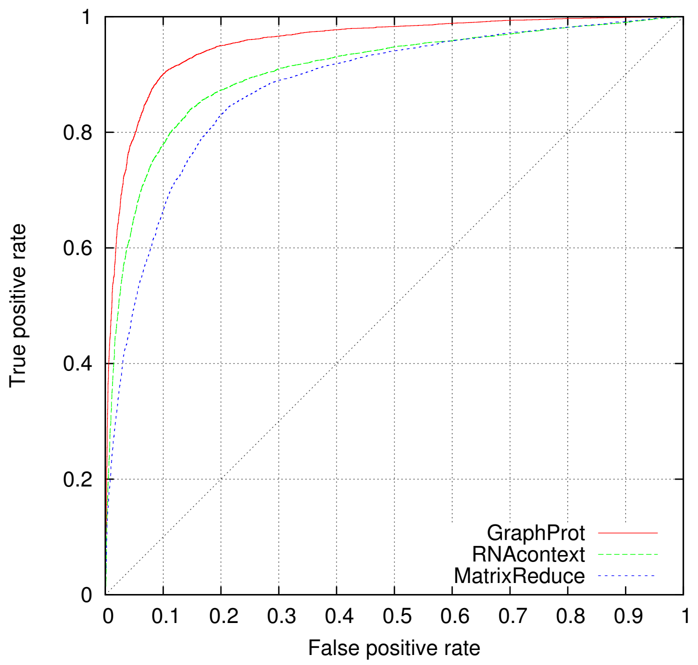

QKI PAR-CLIP

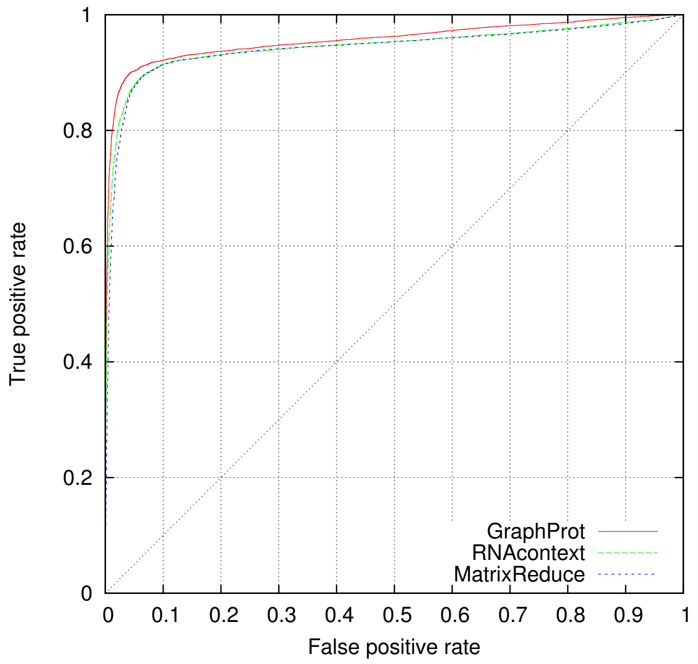

TAF15 PAR-CLIP

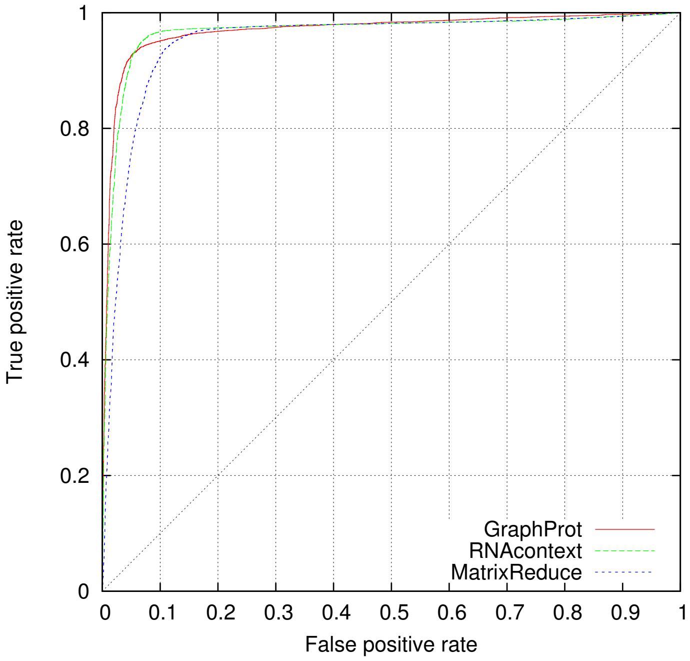

PTB HITS-CLIP

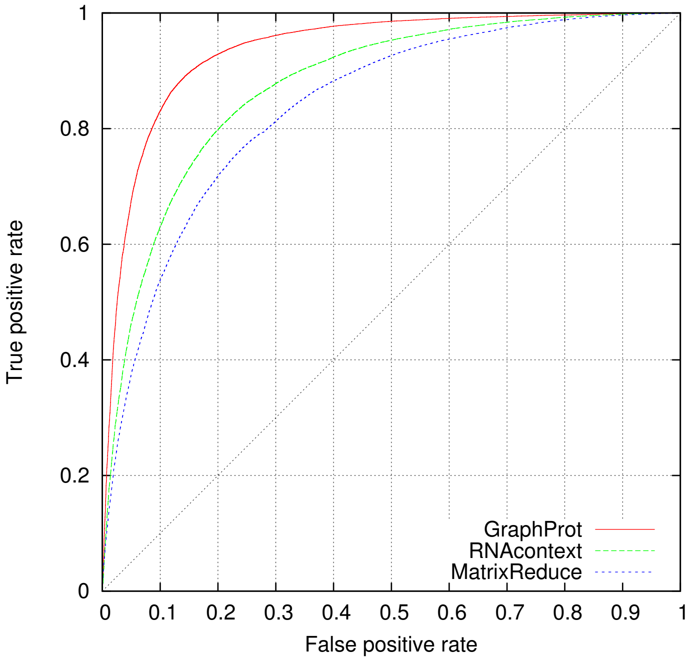

ZC3H7B PAR-CLIP

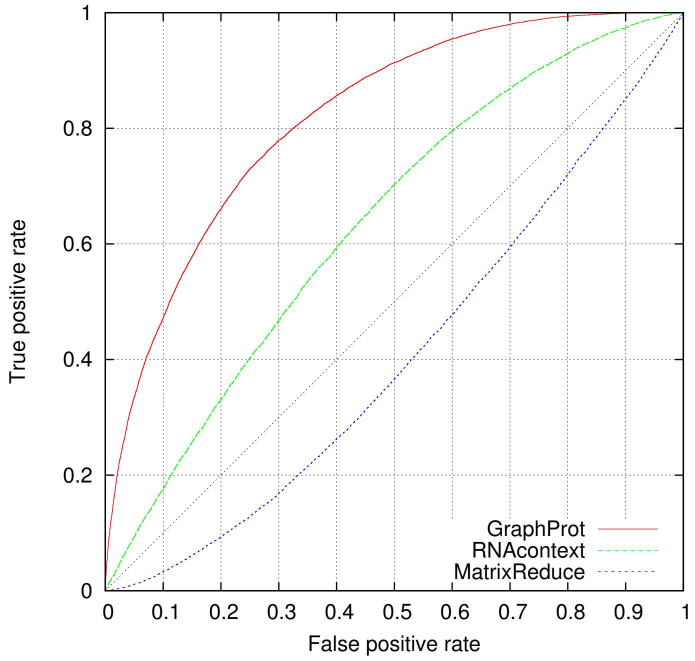

Fusip RNAcompete

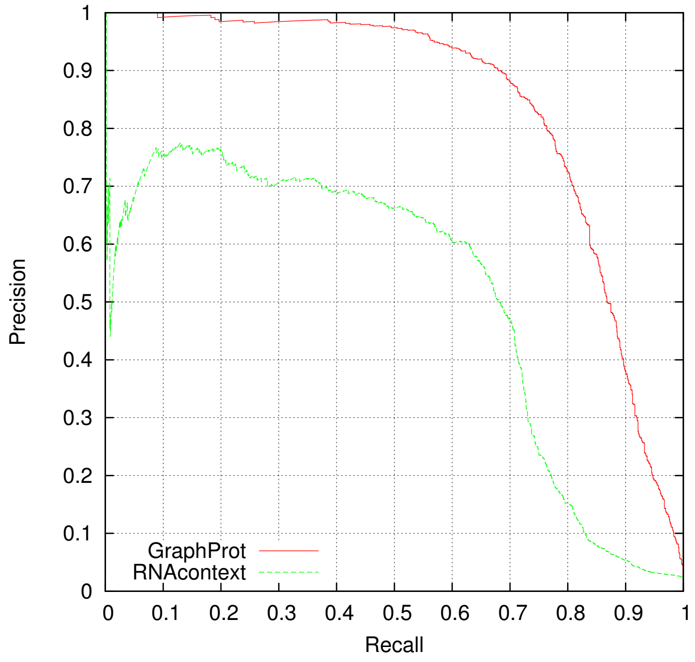

ELAVL1 RNAcompete

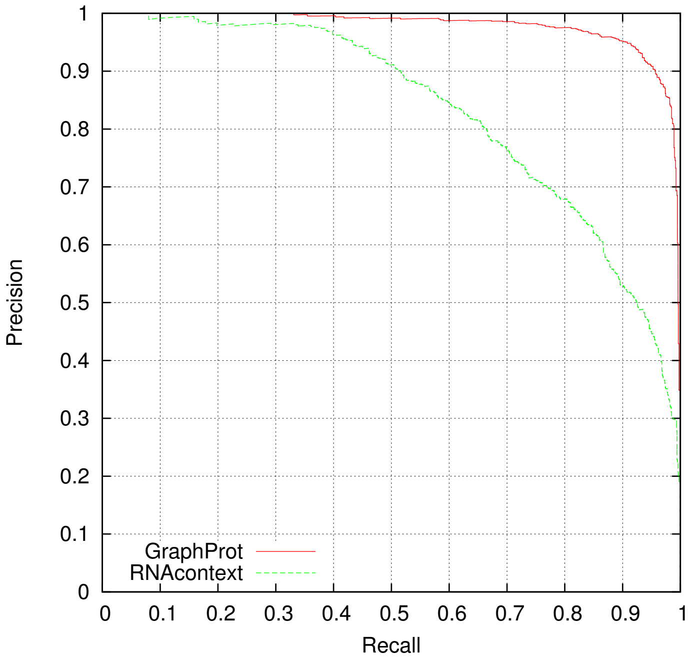

PTB RNAcompete

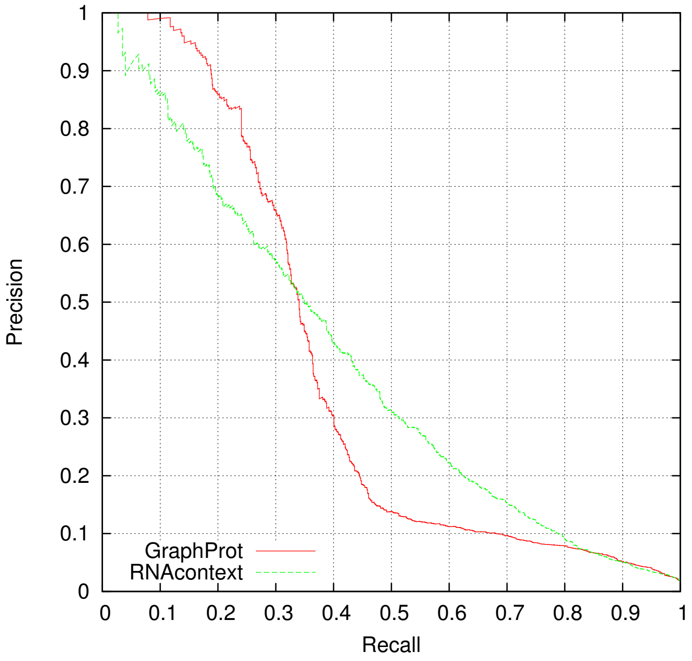

RBM4 RNAcompete

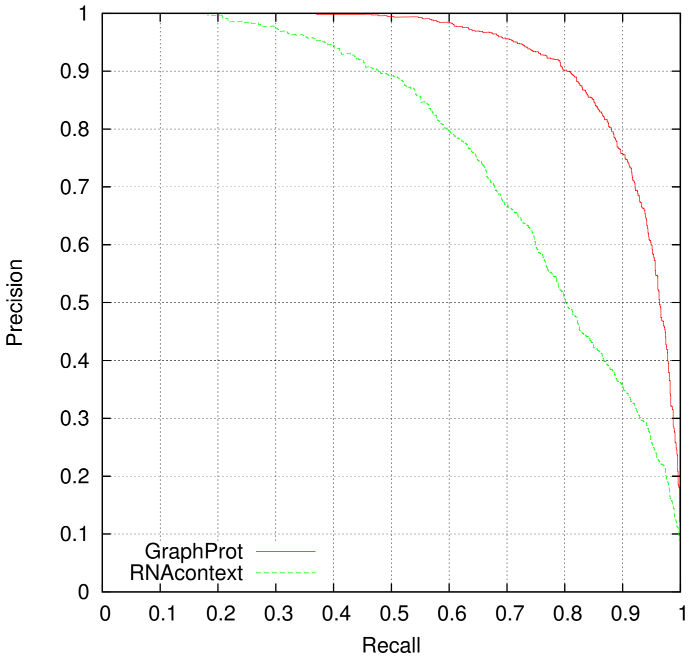

SFRS1 RNAcompete

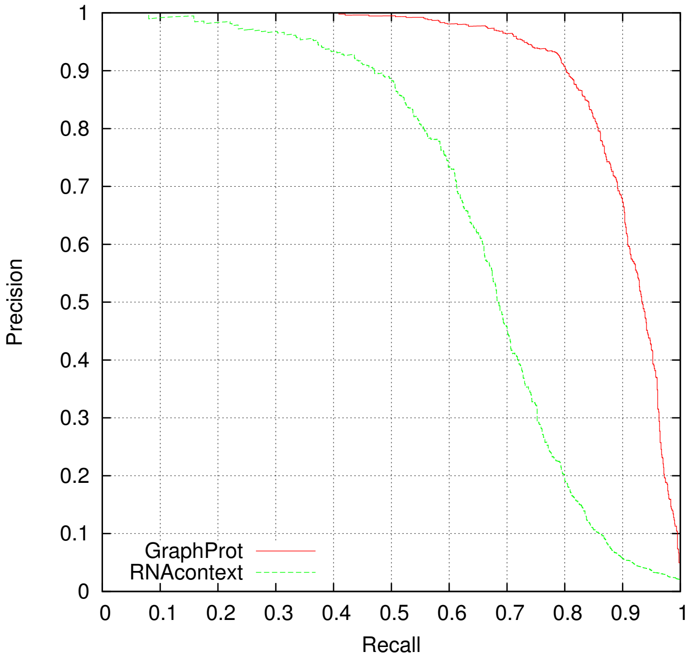

SLM2 RNAcompete

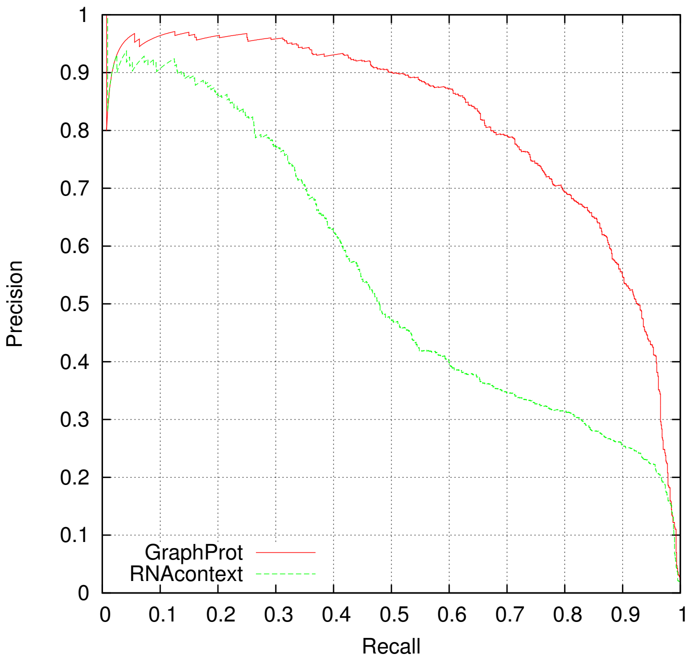

U1A RNAcompete

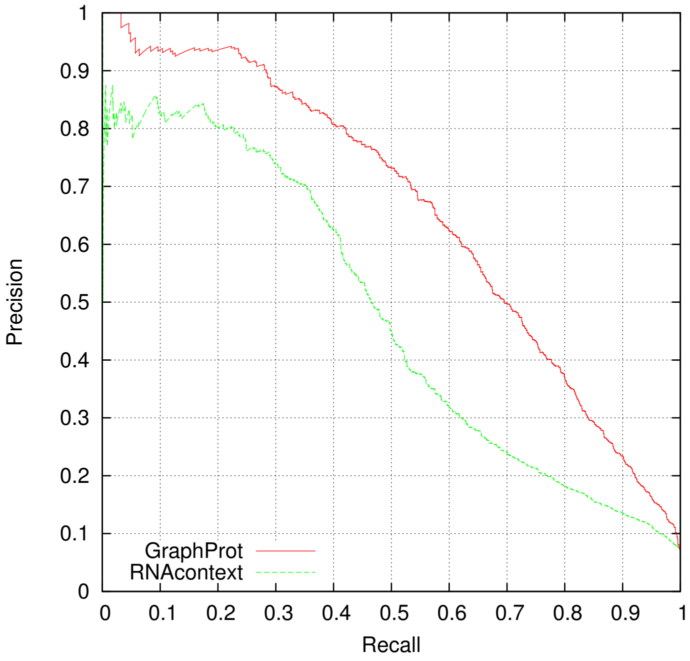

VTS1 RNAcompete

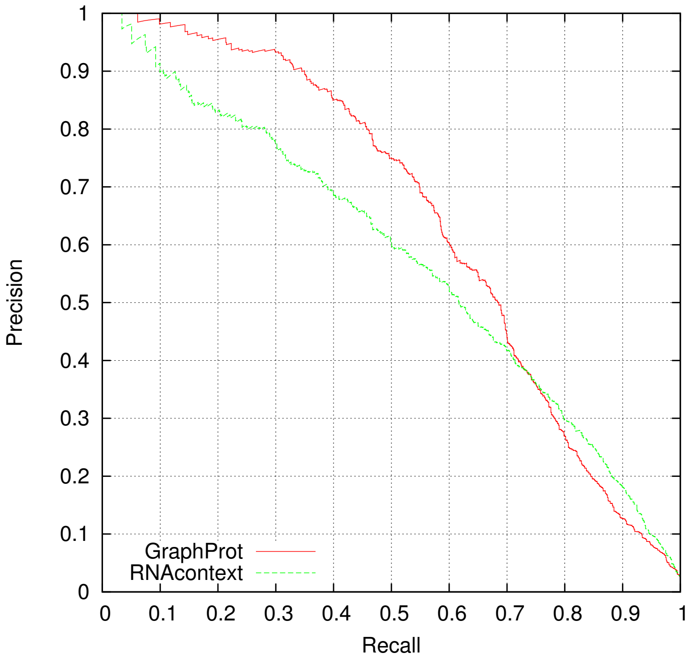

YB1 RNAcompete

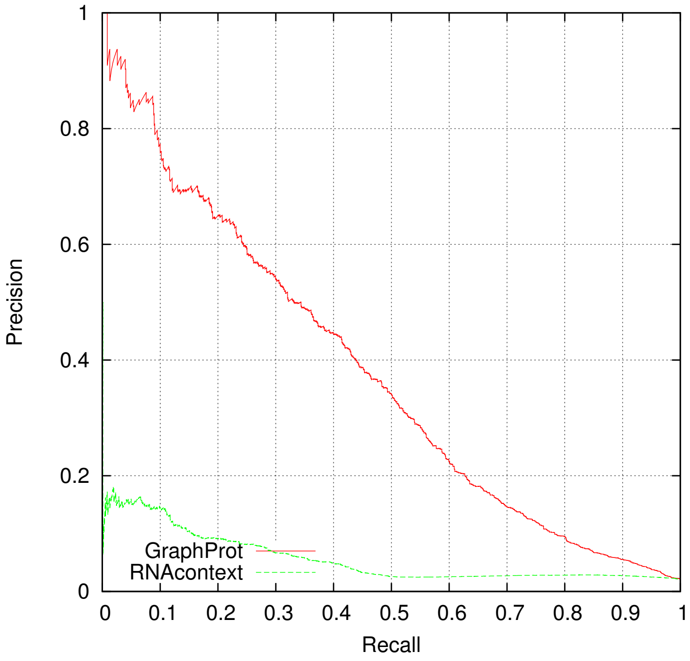

Supplement: Additional file 2 — CLIP cross-validation and RNAcompete validation results (PDF). The file contains results of the CLIP cross-validations and RNAcompete evaluations (AUROC and APR), estimated predictive performance using tenfold cross-validation, receiver operating characteristic curves for the CLIP cross-validations and precision-recall curves for the RNAcompete evaluations. [file gb-2014-15-1-r17-S2.pdf]
