# Supplementary material for: GraphProt: modeling binding preferences of RNA-binding proteins
Source: Genome Biol. 2014 Jan 22;15(1):R17. doi: 10.1186/gb-2014-15-1-r17 (PMC4053806; doi:10.1186/gb-2014-15-1-r17)
Supplement: Additional file 3 — Binding to double-stranded regions (PDF). Binding to double-stranded regions depends on distant stretches of nucleotides involved in the base pairing. [file gb-2014-15-1-r17-S3.pdf]

Binding to double-stranded regions depends on distant stretches of nucleotides involved in the base pairing

Experimental evidence suggests that RBPs show sequence specificity when binding not only to unpaired regions—but also to double-stranded stem regions. Lee *et al.* [Lee et al., 2002] applied SELEX [Tuerk and Gold, 1990] to the C5 protein and identified bound sequences that fold into a verified stem-loop structure (Figure 1) where not only the base pairs are conserved, but also three stretches of nucleotides; both the stem and the loop were essential for C5 binding.

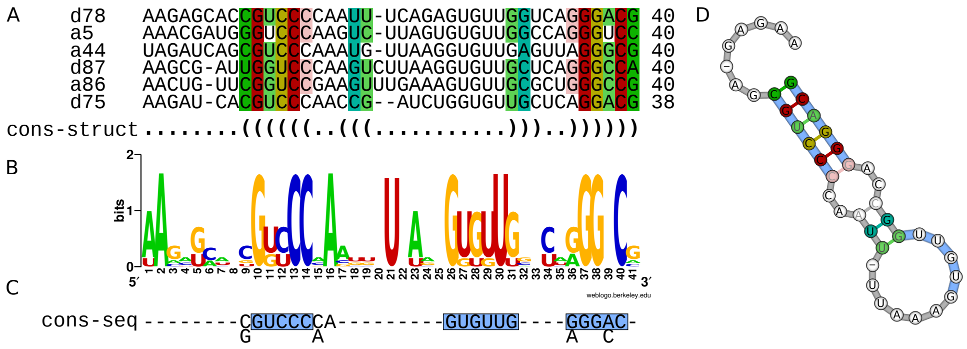

Figure 1: **C5 binds to a stem-loop structure where three disconnected sequence motifs affect binding affinity [Lee et al., 2002].** (A) Sequence-structure alignment of C5-bound aptamers using LocaRNA [Will et al., 2012]. (B) Sequence logo derived from the alignment; it clearly shows disconnected sequence motifs. (C) The Consensus sequence with sequence motifs important for binding from [Lee et al., 2002]; the consensus sequence coincides with the sequence logo in B; (D) Conserved secondary structure; the motifs from the consensus sequence in C are indicated with blue bars. Both the structure and the binding importance of the conserved motifs were verified experimentally.

References

[Lee et al., 2002] Lee, J. H., Kim, H., Ko, J., and Lee, Y. (2002). Interaction of C5 protein with RNA aptamers selected by SELEX. *Nucleic Acids Res*, 30(24):5360–8.

[Tuerk and Gold, 1990] Tuerk, C. and Gold, L. (1990). Systematic evolution of ligands by exponential enrichment - RNA ligands to bacteriophage-T4 DNA-polymerase. *Science*, 249(4968):505–510.

[Will et al., 2012] Will, S., Joshi, T., Hofacker, I. L., Stadler, P. F., and Backofen, R. (2012). LocARNA-P: Accurate boundary prediction and improved detection of structural RNAs. *RNA*, 18(5):900–14.
