# Supplementary material for: GraphProt: modeling binding preferences of RNA-binding proteins
Source: Genome Biol. 2014 Jan 22;15(1):R17. doi: 10.1186/gb-2014-15-1-r17 (PMC4053806; doi:10.1186/gb-2014-15-1-r17)
Supplement: Additional file 4 — Additional analyses for Ago2 binding sites (PDF). Full distributions of Ago2 binding site hits corresponding to Figure 9B and additional analyses on microRNA target prediction corresponding to Figure 9A,B. [file gb-2014-15-1-r17-S4.pdf]

Additional File: Ago2 knockdown analysis

References

[1] Schmitter D, Filkowski J, Sewer A, Pillai RS, Oakeley EJ, Zavolan M, Svoboda P, Filipowicz W: **Effects of Dicer and Argonaute down-regulation on mRNA levels in human HEK293 cells**. *Nucleic Acids Res* 2006, **34**(17):4801–15.

[2] Anders G, Mackowiak SD, Jens M, Maaskola J, Kuntzagk A, Rajewsky N, Landthaler M, Dieterich C: **doRiNA: a database of RNA interactions in post-transcriptional regulation**. *Nucleic Acids Res* 2012, **40**(Database issue):D180–6.

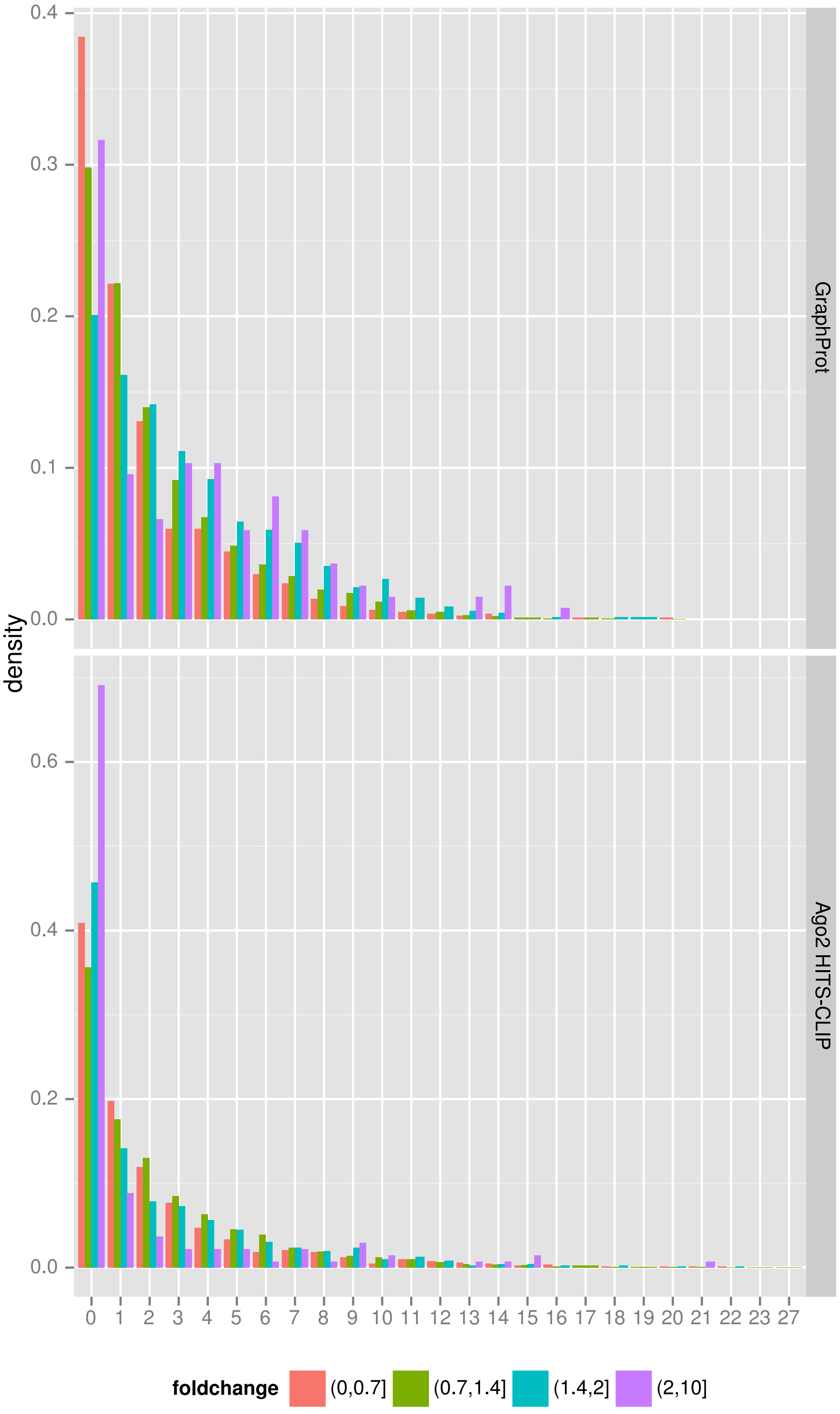

Figure 1: Full distribution of the number of binding-site hits per 3'-UTR as depicted in Figure 9, comparing high-scoring GraphProt predictions and HITS-CLIP sites.

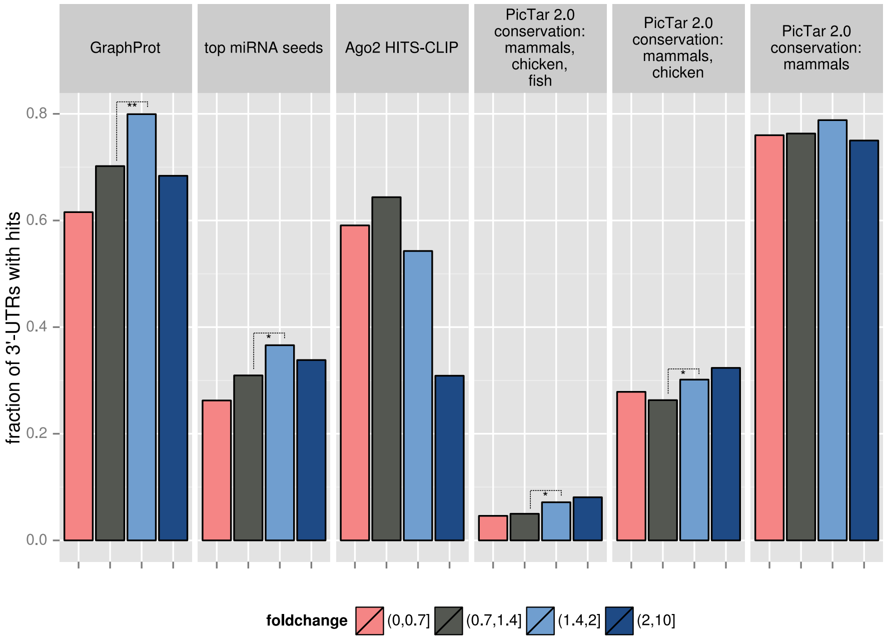

Figure 2: Number of 3'-UTRs with at least one Ago2 binding-site hit. microRNA seed hits ("top miRNA seeds") were calculated for seeds AAAGUGC, GUAAACA and AUAAAGU as described by Schmitter and colleagues [1]. PicTar 2.0 microRNA predictions were downloaded from doRiNA [2]. In all cases, any oberlapping or bookended sites were merged prior to counting. Asterisk indicates statistically significant increase (t-test, *: $p < 0.05$, **: $p < 0.001$).

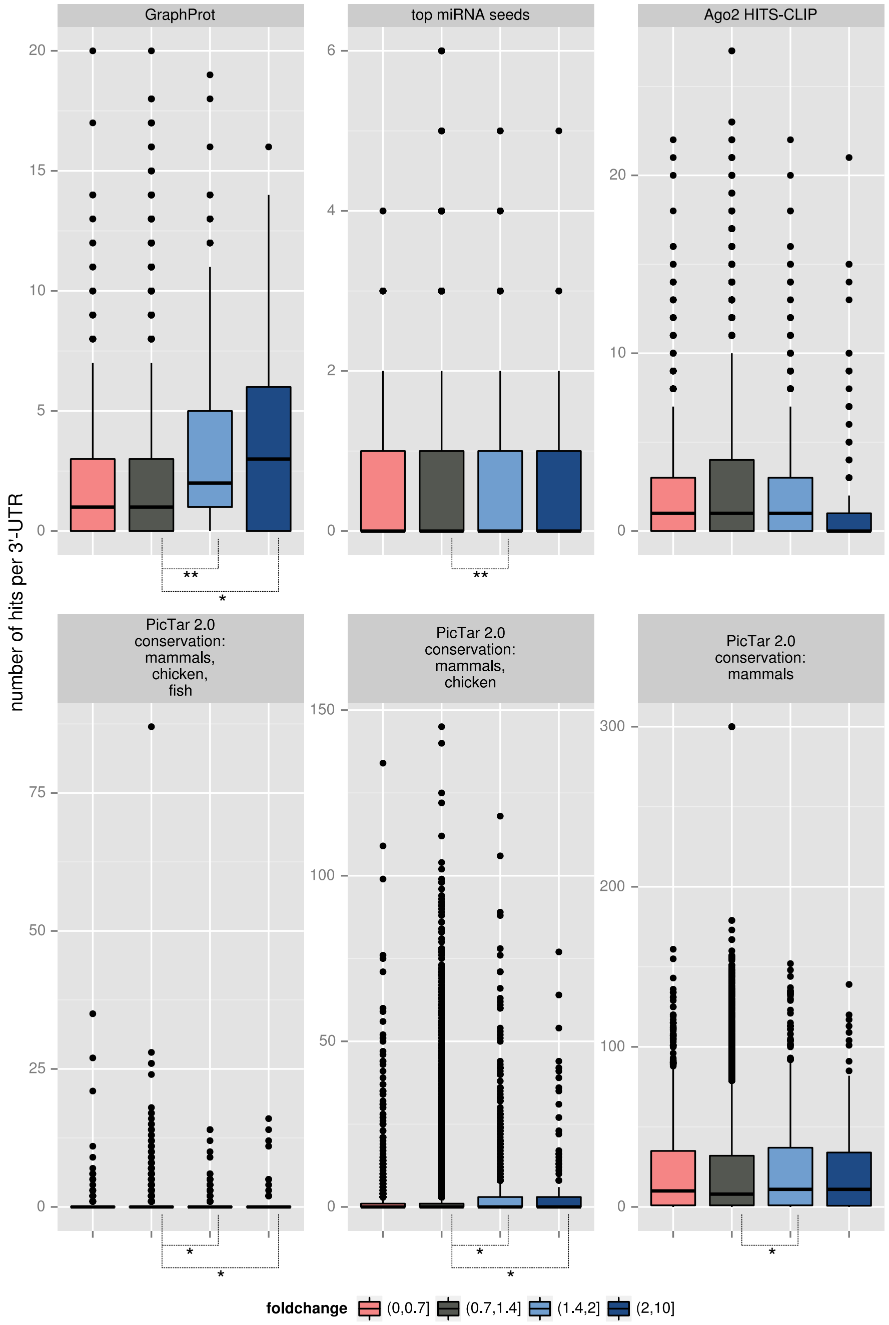

Figure 3: Number of binding-site hits per 3'-UTR. microRNA seed hits ("top miRNA seeds") were calculated for seeds AAAGUGC, GUAAACA and AUAAAGU as described by Schmitter and colleagues [1]. PicTar 2.0 microRNA predictions were downloaded from doRiNA [2]. In all cases, any oberlapping or bookended sites were merged prior to counting. Asterisk indicates statistically significant increase (Wilcoxon rank sum test, *: $p < 0.05$, **: $p < 0.001$).
