# Supplementary material for: GraphProt: modeling binding preferences of RNA-binding proteins
Source: Genome Biol. 2014 Jan 22;15(1):R17. doi: 10.1186/gb-2014-15-1-r17 (PMC4053806; doi:10.1186/gb-2014-15-1-r17)

GraphProt motifs for CLIP-seq models

This document shows sequence and structure motifs for all CLIP-seq sets. Structure motifs are annotated with the full set of structure elements – stems (S), external regions (E), hairpins (H), internal loops (I), multiloops (M) and bulges (B). Accessibility motifs are simplified representations of the full structure motifs and only distinguish – paired (P) and unpaired / accessible nucleotides (U).

ALKBH5 PAR-CLIP

sequence

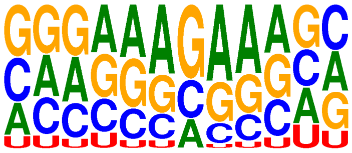

structure

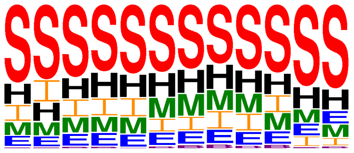

accessibility

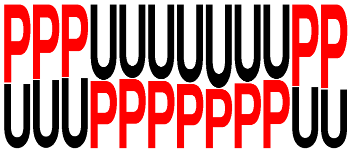

C17ORF85 PAR-CLIP

sequence

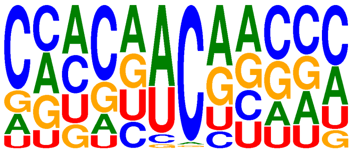

structure

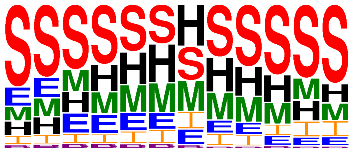

accessibility

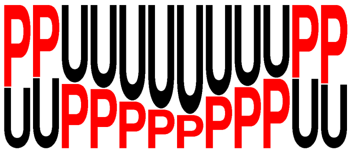

C22ORF28 PAR-CLIP

sequence

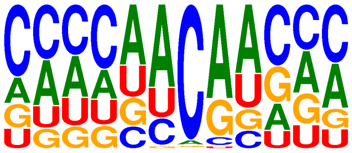

structure

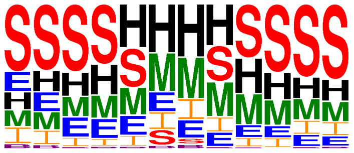

accessibility

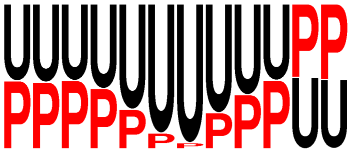

CAPRIN1 PAR-CLIP

sequence | structure | accessibility

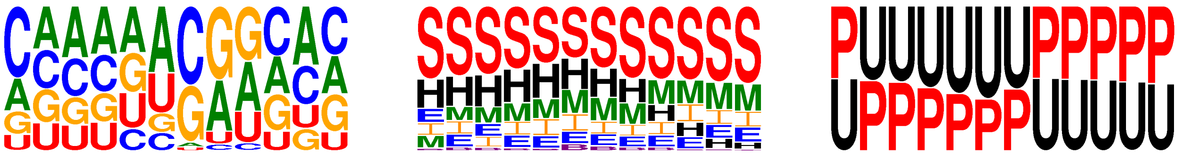

Ago2 HITS-CLIP

sequence | structure | accessibility

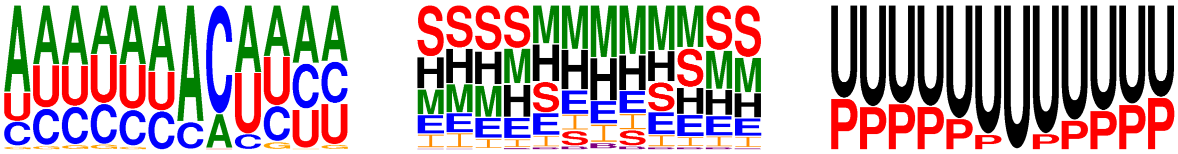

ELAVL1 HITS-CLIP

sequence | structure | accessibility

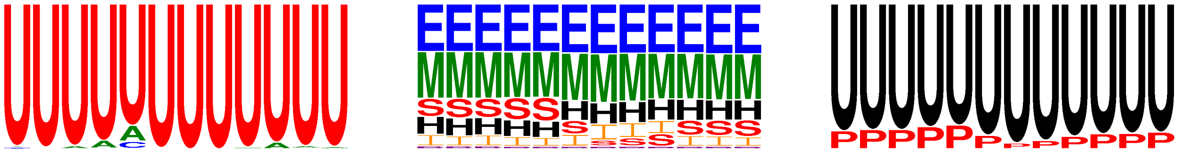

SFRS1 HITS-CLIP

sequence | structure | accessibility

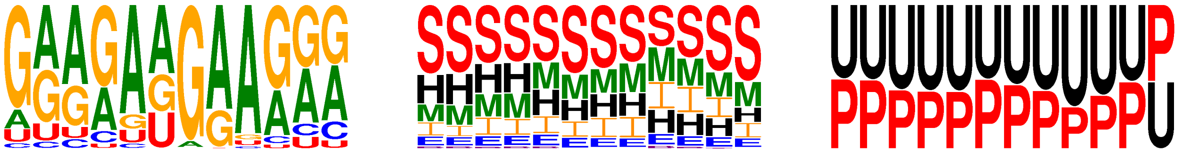

HNRNPC iCLIP

sequence | structure | accessibility

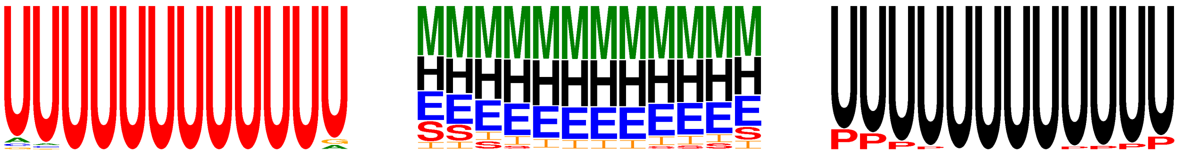

TDP43 iCLIP

sequence structure accessibility

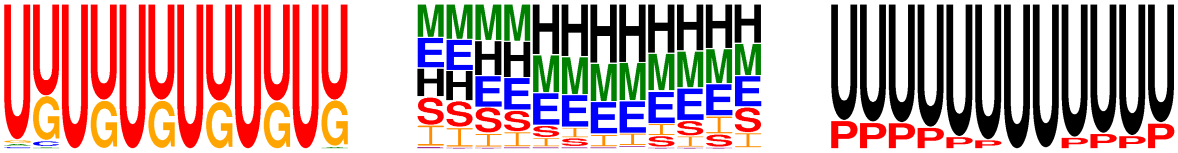

TIA1 iCLIP

sequence structure accessibility

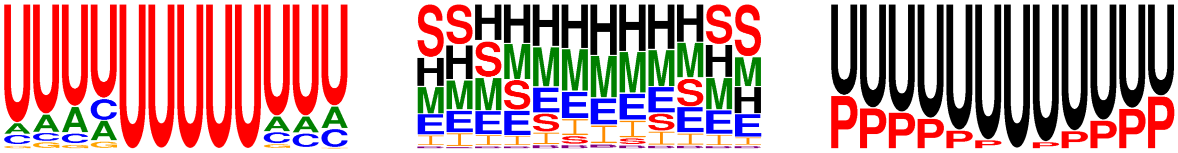

TIAL1 iCLIP

sequence structure accessibility

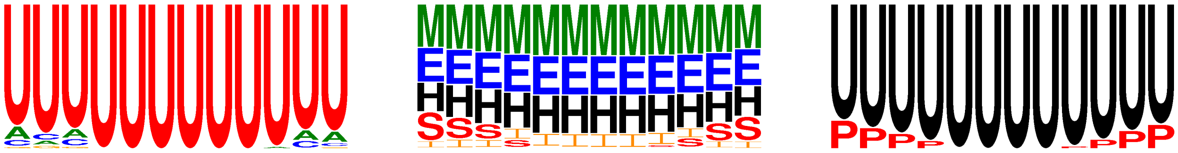

Ago1-4 PAR-CLIP

sequence structure accessibility

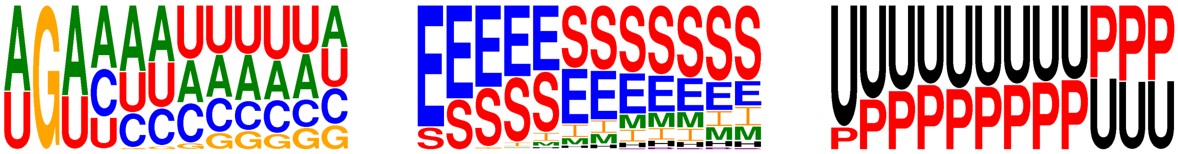

ELAVL1 PAR-CLIP (A)

sequence structure accessibility

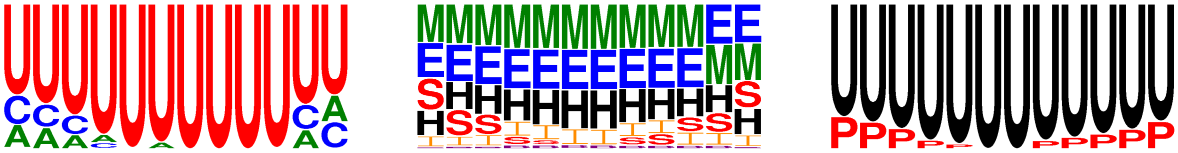

ELAVL1 PAR-CLIP (B)

sequence structure accessibility

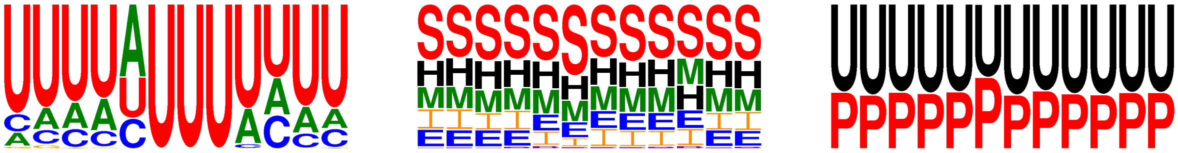

EWSR1 PAR-CLIP

sequence structure accessibility

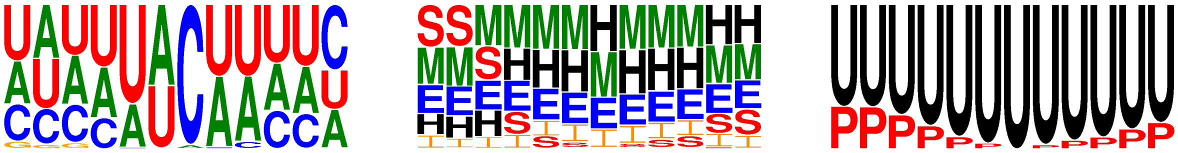

FUS PAR-CLIP

sequence structure accessibility

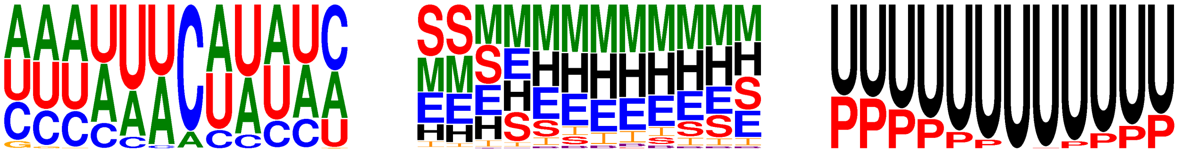

ELAVL1 PAR-CLIP (C)

sequence structure accessibility

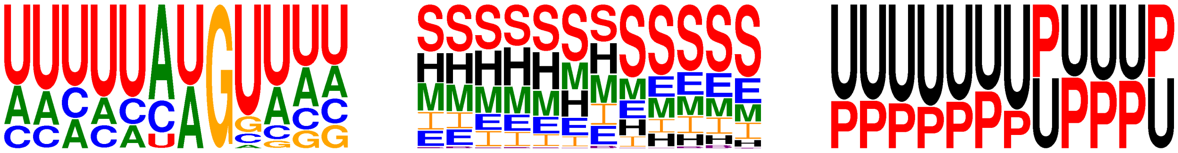

IGF2BP1-3 PAR-CLIP

sequence structure accessibility

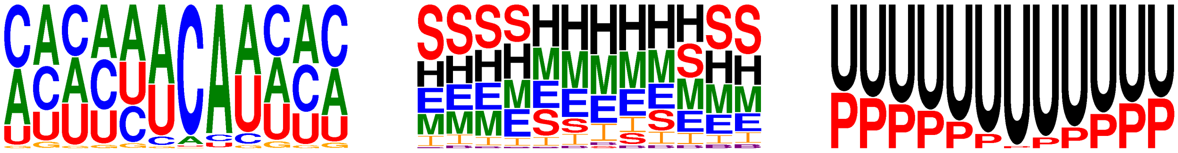

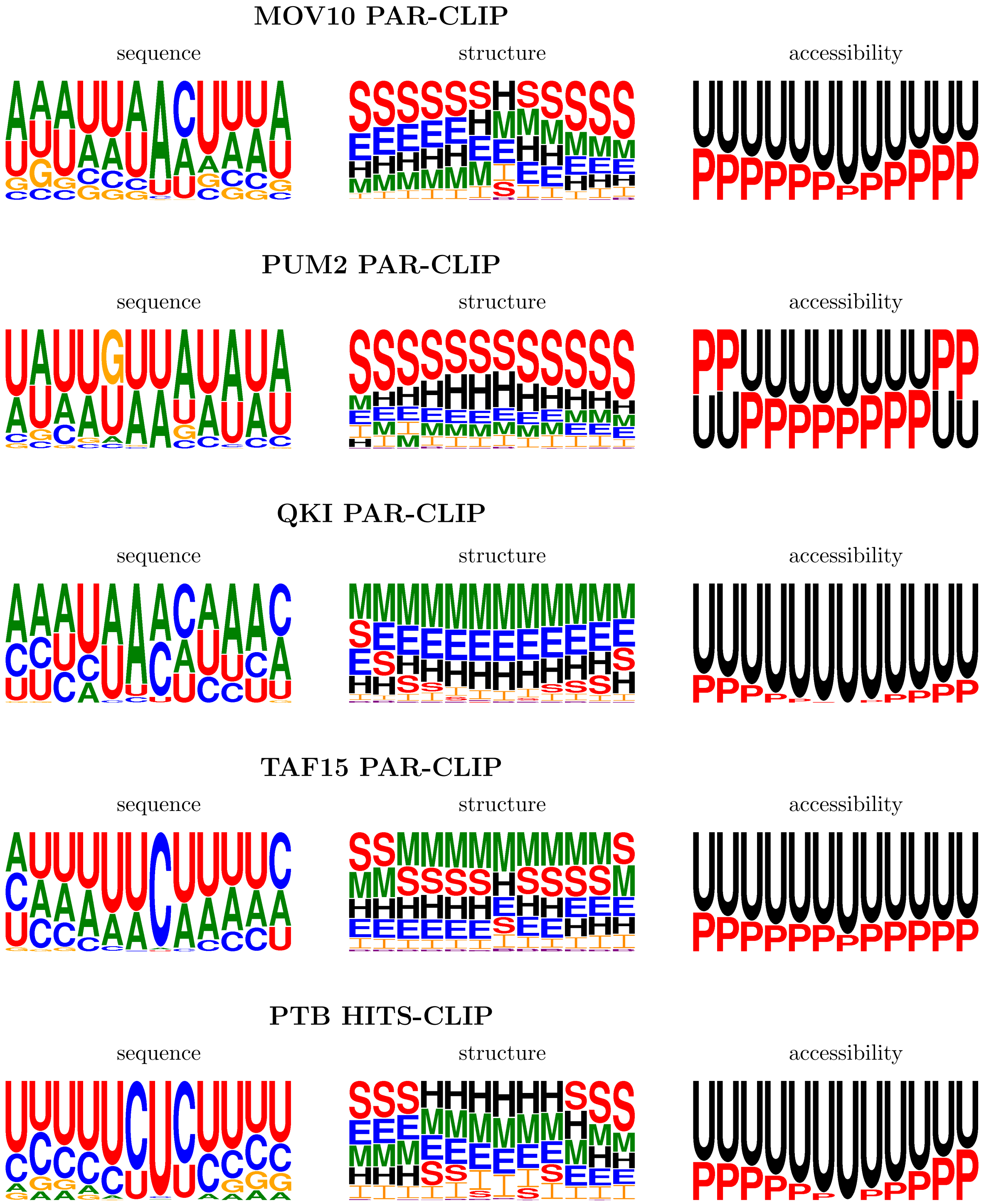
MOV10 PAR-CLIP
sequence
structure
accessibility
PUM2 PAR-CLIP
sequence
structure
accessibility
QKI PAR-CLIP
sequence
structure
accessibility
TAF15 PAR-CLIP
sequence
structure
accessibility
PTB HITS-CLIP
sequence
structure
accessibility

ZC3H7B PAR-CLIP

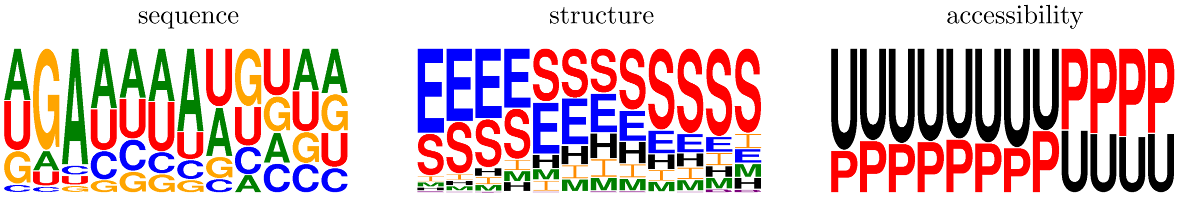

Supplement: Additional file 10 — GraphProt motifs for CLIP-seq models (PDF).GraphProt structure motifs including simplified profiles distinguishing only paired and unpaired positions. [file gb-2014-15-1-r17-S10.pdf]
