# Supplementary material for: GraphProt: modeling binding preferences of RNA-binding proteins
Source: Genome Biol. 2014 Jan 22;15(1):R17. doi: 10.1186/gb-2014-15-1-r17 (PMC4053806; doi:10.1186/gb-2014-15-1-r17)
Supplement: Additional file 11 — GraphProt version 1.0.1 (ZIP). [file gb-2014-15-1-r17-S11.zip › GraphProt-1.0.1/README.html]

# GraphProt Manual

This software package contains the GraphProt framework as published in
“GraphProt: modeling binding preferences of
RNA-binding proteins”.

## Installation

GraphProt contains a precompiled version of “EDeN”, the SVM package used for
feature creation and classification. This binary should run on most Linux-based
systems. In case it does not run on your system, please call
“bash ./recompile\_EDeN.sh” from the GraphProt main directory.

GraphProt uses various opensource software packages. Please make sure that the
follwing programs are installed and accessible via the PATH environment variable
(i.e. you should be able to call the programs by just issuing the command).

- RNAshapes is used for GraphProt secondary structure predictions (recommended version: 2.1.6, http://bibiserv.techfak.uni-bielefeld.de/rnashapes/)
- perf is used to calculate prediction performance (http://osmot.cs.cornell.edu/kddcup/software.html)
- libsvm is used for support vector regressions (http://www.csie.ntu.edu.tw/~cjlin/libsvm/)
- GNU make is used as the pipeline backend (http://www.gnu.org/software/make/)
- R is used to process nucleotide-wise margins for motif creation (www.r-project.org/)
- The R plyr package is required for calculating motifs (http://plyr.had.co.nz/) and can be installed from within R by issuing the command “install.packages(‘plyr’)”.
- GraphProt uses WebLogo3 to plot sequence and structure motifs. GraphProt was tested using version WebLogo 3.2 http://code.google.com/p/weblogo/downloads.

GraphProt will scan for these programs and notify you if something seems amiss.
GraphProt contains a copy of fastapl (http://seq.cbrc.jp/fastapl/index.html.en).

## Usage

GraphProt analyses are started by calling “GrapProt.pl”. If no options are given,
GraphProt.pl will display a help message summarizing all available options.
The default mode is to run analyses in classification setting,
switch to regression setting using the parameter -mode regression.
In general, GraphProt analyses are run by issuing different actions, e.g.

GraphProt.pl –action train -fasta train\_positives.fa -negfasta train\_negatives.fa

GraphProt supports input sequences in fasta format. The **viewpoint** mechanism
sets viewpoints to all nucleotides in **uppercase** letters, nucleotides in
**lowercase** letters are only used for RNA structure predictions.

GraphProt parameters abstraction, R, D, bitsize, c, epsilon, epochs and lambda
are set to default values. For best results, optimized parameters should be
obtained with the ls parameter optimization setting.

Input files in classification setting are specified with parameters “-fasta”
(binding sites) and “-negfasta” (unbound sites). For regressions, input sequences
are specified with “-fasta” and sequence scores with “-affinities”. For each
sequence, the affinity file should contain one value per line.

Output filenames can be specified via a prefix (-prefix); if no prefix is given,
the default is “GraphProt”.

### Available Actions

#### ls - Parameter Optimization

Determines optimized parameters. Parameters are printed to screen and written
to file “GraphProt.param”.

#### cv - Crossvalidation

Runs a 10-fold crossvalidation. Crossvalidation results are written to file
“GraphProt.cv\_results”.

#### train - Model Training

Trains a GraphProt model. The model is written to file “GraphProt.model”.

#### predict - Predict binding for a whole site

Predict binding of whole sequences, e.g. CLIP sites. Margins are written to file
“GraphProt.predictions”. Each line of this file gives the margin for one sequence in the second column,
in the same order as the fasta file. In classification setting the first column contains the class,
in regression setting the first column contains, if specified, the affinities, otherwise
1.

#### predict\_profile - Predict binding profiles

Predict binding profiles (nucleotide-wise margins) for sequences. Nucleotide-wise margins are written
to file “GraphProt.profile”, this file contains three columns:

1. number of sequence
2. number of nucleotide
3. prediction for this nucleotide

Please note that with GraphProt structure models this action currently only supports sequences of up to 150 nt.

#### predict\_has - Predict high-affinity binding sites

Predict high-affinity target sites as showcased in the GraphProt paper.
Selects all regions with average scores within 12nt above a given percentile (parameter -percentile, defaults to 99).
Average nucleotide-wise margins of high-affinity sites are written to file GraphProt.has.
This file contains three columns:

1. number of sequence
2. number of nucleotide
3. average prediction this nucleotide

#### motif - Create RNA sequence and structure motifs

Create RNA sequence and structure motifs as described in the “GraphProt” paper.
Motifs are written to files “GraphProt.sequence\_motif.png” and “GraphProt.structure\_motif.png”.

## Advanced Usage

In addition to the integrated usage via GraphProt.pl, individual tasks such as
creation of RNA structure graphs or calculation of features can be accomplished
using the following tools:

- fasta2shrep\_gspan.pl: graph creation
- EDeN/EDeN: NSPD kernel and SGD support vector machine

Usage information for these tools can be obtained by specifying the “-h” option.

### RNA sequence and structure graphs

RNA sequence and structure graphs are created using fasta2shrep\_gspan.pl. Structure graphs
are created using the following parameters. The user has to chose an appropriate
RNAshapes **ABSTRACTION\_LEVEL**.

fasta2shrep\_gspan.pl –seq-graph-t –seq-graph-alph -abstr -stdout -M 3 -wins ‘150,’ -shift ‘25’ -fasta PTBv1.train.fa -t **ABSTRACTION\_LEVEL** | gzip > PTBv1.train.gspan.gz

RNA sequence graphs are created using the following parameters:

fasta2shrep\_gspan.pl –seq-graph-t -nostr -stdout -fasta PTBv1.train.fa | gzip > PTBv1.train.gspan.gz

### NSPD kernel and SGD support vector machine

For example, 10-fold crossvalidation using EDeN is done via:

EDeN/EDeN -a CROSS\_VALIDATION -c 10 -i PTBv1.train.gspan.gz -t PTBv1.train.class -g DIRECTED -b **BIT\_SIZE** -r **RADIUS** -d **DISTANCE** -e **EPOCHS** -l **LAMBDA**

and setting the appropriate parameters for BIT\_SIZE, RADIUS, DISTANCE, EPOCHS
and LAMBDA.
